# Molecular surveillance of *Plasmodium falciparum* drug-resistance markers in Vietnam using multiplex amplicon sequencing (2000–2016)

**DOI:** 10.1038/s41598-023-40935-7

**Published:** 2023-08-25

**Authors:** Eduard Rovira-Vallbona, Johanna Helena Kattenberg, Nguyen Van Hong, Pieter Guetens, Hideo Imamura, Pieter Monsieurs, Driss Chiheb, Annette Erhart, Bui Quang Phuc, Nguyen Xuan Xa, Anna Rosanas-Urgell

**Affiliations:** 1 Department of Biomedical Sciences, Institute of Tropical Medicine, 2000 Antwerp, Belgium; 2 National Institute of Malariology, Parasitology and Entomology, Hanoi, 10200 Vietnam; 3 Present Address: ISGlobal, Hospital Clínic/Universitat de Barcelona, 08036 Barcelona, Catalonia Spain; 4 Present Address: Vrije Universiteit Brussel, Campus Jette, 1090 Brussels, Belgium; 5 Present Address: UZ Brussel, Centre for Medical Genetics, 1090 Brussels, Belgium; 6 Present Address: Medical Research Council Unit, The Gambia at the London School of Hygiene and Tropical Medicine, Fajara, The Gambia

**Keywords:** Malaria, Parasite genomics, Epidemiology

## Abstract

Emergence and spread of *Plasmodium falciparum* resistance to artemisinin-based combination therapies (ACT) is a major challenge for Greater Mekong Subregion countries in their goal to eliminate malaria by 2030. Tools to efficiently monitor drug resistance beyond resource-demanding therapeutic efficacy studies are necessary. A custom multiplex amplicon sequencing assay based on Illumina technology was designed to target the marker of partial resistance to artemisinin (K13), five candidate modulators of artemisinin resistance, the marker of resistance to chloroquine (*crt)*, and four neutral microsatellite loci. The assay was used to genotype 635 *P. falciparum*-positive blood samples collected across seven provinces of Vietnam and one of Cambodia between 2000 and 2016. Markers of resistance to artemisinin partner-drugs piperaquine (copy number of *plasmepsin*-2) and mefloquine (copy number of *multidrug-resistance 1*) were determined by qPCR. Parasite population structure was further assessed using a 101-SNP barcode. Validated mutations of artemisinin partial resistance in K13 were found in 48.1% of samples, first detection was in 2000, and by 2015 prevalence overcame > 50% in Central Highlands and Binh Phuoc province. K13-C580Y variant became predominant country-wide, quickly replacing an outbreak of K13-I543T in Central Highlands. Mutations in candidate artemisinin resistance modulator genes paralleled the trends of K13 mutants*,* whereas resistance to piperaquine and mefloquine remained low (≈ 10%) by 2015–2016. Genomic tools applied to malaria surveillance generate comprehensive information on dynamics of drug resistance and population structure and reflect drug efficacy profiles from in vivo studies.

## Introduction

Artemisinin derivatives (ART) administered in combination with a partner antimalarial drug (ART-based combination therapies, ACT) are the current first-line treatment for uncomplicated *Plasmodium falciparum* malaria worldwide^1^. However, the high-rate of failures after ACT treatment reported in the Greater Mekong Subregion (GMS) challenges malaria elimination in the region and poses a potential threat for malaria control if it expands to other endemic areas^2^. Monitoring drug resistance is thus of paramount importance for National Malaria Control Programs worldwide. The gold standard for drug resistance surveillance is standardized therapeutic efficacy clinical studies, which are costly and logistically challenging in countries with low endemicity -or in those moving towards elimination-, due to the difficulty to enroll sufficient cases in the study period^2,3^. With advances in both elucidation of molecular markers of resistance and access to sequencing technology, genomic tools offer the possibility to increase geographical scale and sample size of surveillance studies without the complex conditions required for clinical trials, as well as detect early signs of antimalarial resistance^4–7^. In this article, we present the use of a custom multiplex amplicon sequencing approach for surveillance of resistance to drugs in ACT formulations and chloroquine in a collection of *P. falciparum* samples from different locations in Vietnam spanning 17 years.

Reduced susceptibility of *P. falciparum* to ART was first reported in Western Cambodia in 2008 and has since spread across GMS^8–10^. It is characterized by delayed clearance of parasites from peripheral blood (i.e. remaining parasitemia 72 h after treatment initiation or a parasite clearance half-life > 5 h) and therefore represents a partial resistance, mainly affecting the ring-stage parasite forms circulating in peripheral blood. Ten non-synonymous mutations in the propeller domain of *kelch13* gene (K13) have been validated as markers of artemisinin partial resistance (ART-R) according to World Health Organization (WHO; namely, F446L, N458Y, M476I, Y493H, R539T, I543T, P553L, R561H, P574L and C580Y)^2,11^. Independent emergence of C580Y and R561H has also occurred in Central America^12,13^ and South-Saharan Africa^14–16^, respectively. In GMS, K13 mutants are more frequently associated with parasite genetic backgrounds containing mutation V127M in *apicoplast ribosomal protein S10* (*arps10*-V127M), D193Y in *ferredoxin* (*fd-*D193Y), T484I in *multidrug-resistance protein 2* (*mdr2-*T484I), and N326S in *chloroquine resistance transporter* (*crt-*N326S)^17–19^. Although efficacy of common ACT formulations -such as dihydroartemisinin-piperaquine (DHA-PPQ) or artesunate-mefloquine (AS-MQ)-remains high in most of the countries where ART-R has been reported, co-emergence of resistance to the ACT-partner drug is a threat for current treatment policies. Resistance to PPQ is associated with multiple gene copies in the *plasmepsin-2 (pm2)* and *-3* gene cluster^20,21^, but also with mutation E415G in *exonuclease* (exo415)^21^ and variants H97Y, F145I, and I218F in *crt*^10,22–24^. Indeed, the expansion of a multidrug resistant lineage combining K13-C580Y with *pm2* amplification^10,25^ (KEL1/PLA1) was likely responsible for high treatment failure rates to DHA-PPQ in Cambodia and Vietnam^26,27^. Resistance to mefloquine is associated with multiple copies of *mdr1* gene^28^, and double K13/*mdr1* mutants have been linked to treatment failure increases in Thailand and Myanmar^29^.

Chloroquine is not used anymore for the treatment of *P. falciparum* but is still widely used in the GMS as first-line treatment for uncomplicated malaria caused by *Plasmodium vivax*. Resistance to chloroquine in *P. falciparum* is attributed to K76T codon change in *crt* together with mutations in nearby codons 73–76, forming the resistant haplotype CVI[E/D]T^30^, which remains highly prevalent in the GMS^6,31^. Strains with CVI[E/D]T background accompanied by mutations in *mdr1* such as Y184F, can modulate susceptibility of parasites to PPQ, mefloquine or lumefantrine^31^.

Vietnam aims to eliminate malaria by 2030^32^. Malaria cases have decreased by 90% between 2000 and 2019—despite occasional resurgences^33,34^, and currently ≈ 80% of all cases occur in forested areas of Central Highlands provinces, populated by ethnic minorities with cross-border mobility with Cambodia^35^. Controlling the expansion of drug resistant lineages is critical to sustain gains in malaria burden reduction. In this study, we developed a custom multiplex amplicon sequencing approach for high-throughput screening of resistance markers for surveillance purposes. The assay, combined with qPCR CNV analysis and data from in vivo therapeutic efficacy trials, was used to investigate the history of emergence and dynamics of resistance to ACT in Vietnam, using samples from eight malaria-endemic provinces collected during a 17-year period (2000–2016).

## Methods

### Sample collection and preparation

Blood samples with confirmed or suspected *P. falciparum* infection were selected from NIMPE/NMCP routine dried blood spot (DBS) collections at malaria sentinel sites across Vietnam, or from research studies conducted by ITM and NIMPE between September 2000 to September 2016 (DBS, whole blood microtainers or DNA aliquots at − 20 °C)^36–40^. A total of 946 candidate samples were identified. Samples with unclear information on location and/or date of collection and those with a reported parasite density of < 250 parasites/μl by microscopy were excluded (see Supplementary Fig. S1). DNA was extracted from three 5 mm punches of DBS or from 200 μl whole blood using the FavorPrep™ 96-well Genomic DNA kit (Favorgen) following manufacturer’s instructions, and a final elution in 200 μl nuclease-free water. Samples without confirmed *P. falciparum* diagnosis were screened by *varATS*-qPCR^41^, and excluded if they tested negative or if Ct > 35. Samples with sufficient DBS left -or DNA in case DBS was not available- were shipped for external genotyping at Wellcome Sanger Institute (Cambridge, UK; see Supplementary Methods) as part of MalariaGEN SpotMalaria Project (https://www.malariagen.net/projects/SpotMalaria; see Supplementary Fig. S1).

Control DNAs were obtained from *P. falciparum* lab strains 3D7 (reference wild-type strain, maintained in continuous culture at ITM), Dd2 (MRA-150, BEI Resources, NIAID, NIH, contributed by David Walliker), CamWT_C580Y (MRA-1251, BEI Resources, NIAID, NIH, contributed by David A. Fidock) and IPC4912 (MRA-1241, BEI Resources, NIAID, NIH, contributed by Didier Ménard), and from non-infected human white blood cells.

### Multiplex amplicon sequencing

Detailed procedures for parasite genotyping using next-generation sequencing are provided in Supplementary Methods, including a step-by-step lab protocol. Briefly, DNA samples were first enriched for *P. falciparum* DNA using selective whole genome amplification (sWGA) adapted from Oyola et al.^42^. Selective WGA products were purified using AMPure XP magnetic beads (Beckman Coulter) and quantified using a Qubit fluorometer (Invitrogen).

The custom oligonucleotide probe panel was designed for the sequencing chemistry of Illumina’s TruSeq Custom Amplicon (*pf*TSCA) and included 21 amplicons targeting 11 different genes: K13 as the marker of ART-R (full-length gene), *crt* codons 72–76 as the marker of chloroquine resistance^43^, *mdr1* codon Y184F as a modulator of resistance to multiple antimalarials^31,43^, candidate genetic modulators of ART-R *arps10*-V127M, *fd*-D193Y, *crt*-N326S, MAL10: 688956 and MAL13/RAD5-homolog-S1158A^18,44–46^, and four microsatellites for parasite genetic diversity (*poly-α*, ARAII, TA81 and *pk2*; see Supplementary Table S1, Supplementary Fig S2). Libraries were prepared from sWGA amplified DNA using TruSeq Custom Amplicon Low Input Library Prep Kit (Illumina), following manufacturer’s guidelines for 96 samples (see Supplementary Methods). All libraries were quantified using KAPA kit for LightCycler 480 (Roche) before pooling. Library denaturation and sequencing was conducted at Centre for Medical Genetics (University of Antwerp, Edegem, Belgium), using a MiSeq instrument and MiSeq Reagent Kit v2 (Illumina) for paired-end sequencing of 2 × 150 bp reads.

Sequence data for 101-SNP barcode^47^ was generated at Wellcome Sanger Institute and provided as nucleotide sequences within a Genetic Report Card (see Supplementary Methods).

### Sequence data analysis

Demultiplexing, alignment and variant calling were performed using TruSeq Amplicon Workflow in MiSeq Reporter software (Illumina), with *P. falciparum* 3D7 build 29 (PlasmoDB) as reference genomce and a variant filter quality cut-off score of 30. A read depth cut-off was set as the maximum number of mapped reads for each amplicon found in negative controls. Mutations were only reported if detected in at least two samples, or if the read count for the alternative allele was above the read cut-off for that amplicon. Loci with missing calls in more than 50% of all samples and controls were excluded. Haplotypes were built from calls with a ≥ 75% within-sample allele frequency to minimize risk of confounding by complex infections. Two amplicons of the panel (*k13.i* and *MAL10*) were sequenced in less than 50% of positive controls and were excluded from further analysis (see Supplementary Table S2). Median read depth of positive controls for the remaining valid amplicons was 7074× (medians range 34–27,805×; Supplementary Table S2). The minimum proportion for minor allele calling was estimated using parasite mixes and set at 0.2 for all markers (see Supplementary Methods, Supplementary Fig. S3).

Complexity of infection (COI; i.e. the estimated number of genetically distinct parasites within the infection) was determined from both microsatellites and SNP barcodes (see Supplementary Methods). Infections were categorized as single clone (COI = 1) vs. multiple clone infections (COI ≥ 2), if 2 microsatellite alleles were found for one or multiple microsatellite markers. COI from SNP barcodes was determined using The Real McCOIL program^48^. Metrics of genetic diversity He (expected heterozygosity) and F_ST_ (genetic differentiation) were calculated using the R packages adegenet and diveRsity, respectively^49,50^. Clustering analysis was performed using both unsupervised (Principal Component Analysis, PCA) and supervised population analysis (Discriminant Analysis of Principal Components, DAPC; see Supplementary Methods). Both microsatellite data from *pf*TSCA and the SNP barcode was combined to achieve the highest possible resolution.

### Copy number of *pm2* and *mdr1* by qPCR

Copy number of *plasmepsin-2* (*pm2*) and *mdr1* were determined by qPCR using the original DNA eluate. Reactions were set-up in a LightCycler480 (Roche) with Power SYBR Green master mix (ThermoFisher), using previously published primers and *ubiquitin conjugated enzyme* (*uce*) as reference gene^20,51^. Amplification efficiencies were calculated using a 7-point tenfold dilution of genomic DNA from 3D7 (94.1% for *pm2*, 93.9% for *mdr1* and 92% for *uce*). Copy numbers were calculated using ddCt method and 3D7 as calibrator sample (one copy of both *pm2* and *mdr1*). Samples with CNV > 1.5 were considered gene amplifications.

### Definitions and statistical analysis

Samples were grouped by region and year of collection for analytical purposes. Three regions were defined based on the geographical location and epidemiological characteristics of each province (see Supplementary Table S3). In brief, Region 1 included Central provinces, characterized by high endemicity, high Day 3 positivity after ACT but moderate treatment failure rates; Region 2 included provinces in South-Central Coast with low malaria risk, moderate Day 3 positivity and low treatment failure rates (i.e., < 10%); finally, Region 3 corresponded to Binh Phuoc province, accounting for the highest Day 3 positivity and treatment failure rates in the country. By time of collection, samples before 2010 was divided in two groups, 2000–2005 and 2006–2010, whereas those > 2010 were divided in three biannual groups (2011–2012, 2013–2014 and 2015–2016) for increased resolution on recent changes in circulating markers. Validated markers of partial artemisinin resistance consisted of 10 mutations in K13 according to WHO list from 2020^2^. Differences in allele frequency by time or region were evaluated using Chi2 or Fisher’s exact test.

### Ethics statement

Blood samples were collected after written informed consent was obtained from the patient or their parents/guardian. Ethical approvals including secondary use of samples were obtained from ethics committees at National Institute of Malariology Parasitology and Entomology (NIMPE, Hanoi) and Ministry of Health (Hanoi) in Vietnam; and from Institute of Tropical Medicine (ITM, Antwerp) and Antwerp University Hospital (UZA, Antwerp) in Belgium. All procedures involving human subjects were performed in accordance with the Declaration of Helsinki. Clinical trials contributing with samples to the present analysis were registered at ClinicalTrials.gov under identifiers NCT01775592 and NCT02604966.

## Results

### Sample selection and performance of *pf*TSCA

**Figure 1:**
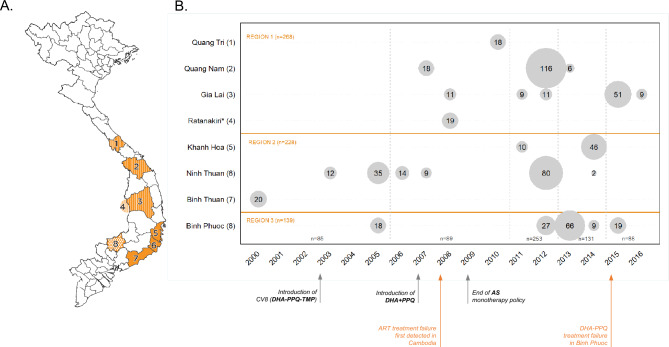
Origin of the samples. (**A**) Map of Vietnam with the location of administrative provinces where samples were collected. Three main geographical regions were defined for analytical purposes based on geographical location and malaria epidemiology criteria (‘Region 1’, stripes; ‘Region 2’, plain orange; ‘Region 3’, dots). Note that Ratanakiri is an administrative province of Cambodia. (**B**) Detail on sample size per location and year. Circles are proportional to sample size. Time groups used for analysis are indicated by vertical dashed lines. The bottom panel indicates relevant events regarding antimalarial treatment policy in Vietnam and Greater Mekong Subregion. This map was developed for the purpose of this article using QGIS 3.10.

Out of the 946 samples available, 635 (67%) had confirmed *P. falciparum* infection and complete records on location and time and were processed in *pf*TSCA workflow (see Supplementary Fig. S1). Fresh DNA extractions were performed on 545 samples originally collected as DBS (545/635, 85.9%), and on 60 samples originally collected as blood in EDTA Vacutainer tubes (60/635, 9.4%); the remaining 30 samples (30/635, 4.7%) were frozen aliquots of previously extracted DNA. Samples originated from seven Vietnamese provinces (n = 616) or from an area in the Cambodian province of Ratanakiri within a 6 km radius of Vietnam’s border (n = 19; Fig. 1).

**Figure 2:**
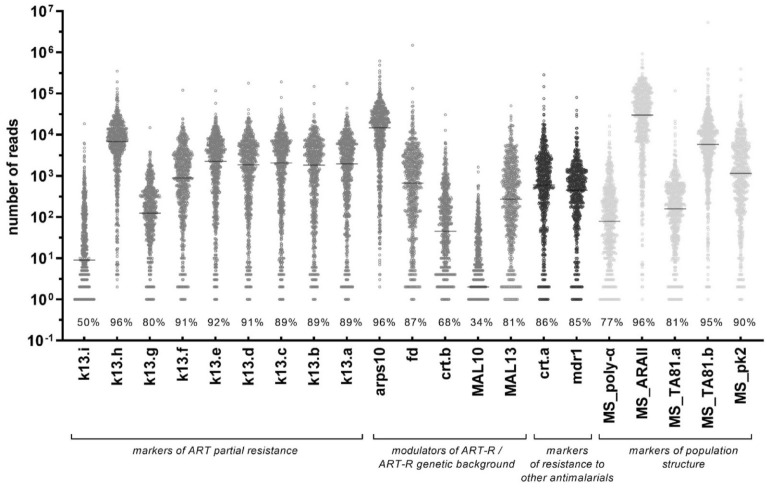
Read depth for amplicons in the *pf*TSCA assay. The name of each amplicon corresponds to the gene target followed by a letter in genes that require multiple amplicons to cover the target region. Horizontal lines indicate median read count. Values (%) indicate the proportion of samples with reads above the read depth cut-off for each amplicon. MS, microsatellite.

The mean number of samples with valid read counts for amplicons in *pf*TSCA was 86.0% (means range 68.7–98.1%), and the median read depth was 1561x (medians range 142–36,281×; Fig. 2, Supplementary Table S4). The number of sequenced amplicons per sample increased with parasite density, irrespective of quantification method (microscopy or qPCR) or collection method (DBS or as whole blood pellet from venipuncture, see Supplementary Fig. S4). External genotyping of 529 samples conducted at Wellcome Sanger Institute showed an agreement of > 95.6% with *pf*TSCA results for variant detection at any frequency, and > 94.1% for the identification of the major allele (see Supplementary Methods, Supplementary Table S5, Supplementary Data S1).

### Genetic markers of resistance to ACT in Vietnam (2000–2016)

#### Markers of ART-R

**Figure 3:**
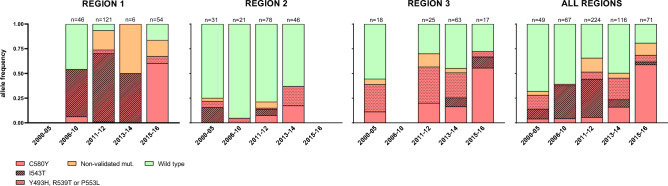
Frequency of K13 mutations as markers of artemisinin partial resistance in Vietnam (2000–2016). Bar charts indicate the percentage of samples with K13 validated mutations (red), non-validated mutations after codon 440 (orange), and wild-type parasites (green). Specific codon changes for validated mutations are indicated with different fill patterns. Data is shown by region and years. Region 1: Quang Tri, Quang Nam, Gia Lai and Ratanakiri (Cambodia) provinces; Region 2: Khanh Hoa, Ninh Thuan, and Binh Thuan provinces: Region 3: Binh Phuoc province.

A total of 13 different non-synonymous K13-allelic variants were identified in 327 samples (327/610, 53.6%). K13 SNPs included ART-R validated mutations Y493H, R539T, I543T, P553L and C580Y as well as previously unreported codon changes G484V and P443L (see Supplementary Table S6, Supplementary Data S1). Among samples with the whole K13 propeller domain sequence (i.e., valid sequences for amplicons from *k13.f* to *k13.a*; n = 526), 56.5% (296/526) carried a non-synonymous mutation and in 48.1% (252/526) this was a validated marker of ART-R (Fig. 3). ART-R validated mutations increased from 28.6% in the 2000–2005 period to 69% in 2015–2016 period (p < 0.001, Chi2 test), and was highest in Region 1 followed by Region 3/Binh Phuoc (red-colored bars in Fig. 3). Frequency of K13 validated mutations was lowest in Region 2 for all time periods, with provincial prevalence of 10.5% (13/124) in Ninh Thuan (data until 2012) and 38.3% (18/47) in Khanh Hoa (data until 2014). Most common ART-R mutations were I543T (first detected in 2 samples from Binh Thuan in year 2000 and the predominant mutation until 2013), and C580Y (first detected in 2005 and the predominant across all regions by 2015; Fig. 3, Supplementary Data S1).

#### Genetic background of ART-R

**Figure 4:**
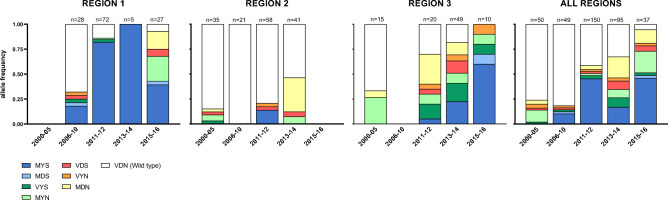
Frequency of ART-R genetic background alleles in Vietnam (2000–2016). Bar charts show the frequency of haplotypes constructed from the combination of *arps10*-V127M, *fd*-D193Y and *crt*-N326S. Reference haplotype VDN is shown in white and mutant haplotypes at either one, two or all three positions are colored. Data is shown by region and years. Region 1: Quang Tri, Quang Nam, Gia Lai and Ratanakiri (Cambodia) provinces; Region 2: Khanh Hoa, Ninh Thuan, and Binh Thuan provinces: Region 3: Binh Phuoc province.

Three out of the four major mutations previously associated with a parasite genetic background (PGB) of ART-R in the GMS were successfully genotyped in the multiplex *pf*TSCA assay (*arps10-*V127M*, fd-*D193Y and *crt-*N326S; but not *mdr2*-T484I). A steady increase in frequency of mutations at all three PGB loci was observed in the study period (Fig. 4, Supplementary Fig. S5). The most common haplotype was the *arps10/fd/crt* triple mutant MYS, and it was significantly associated with presence of K13 mutations (p < 0.001). Parasites with mutations at one or more of PGB markers represented > 75% of the parasite population in Region 1 (Central Highland) by 2011. On the contrary, the frequency of PGB mutants was low in Region 2 (coastal provinces). The MAL13/RAD5-homolog-S1158A allele, a marker previously associated with both delayed clearance and increased survival in RSA^44–46^ was almost fixed at 99.0% (511/516; see Supplementary Data S1).

#### Markers of resistance to partner drugs piperaquine and mefloquine

**Figure 5:**
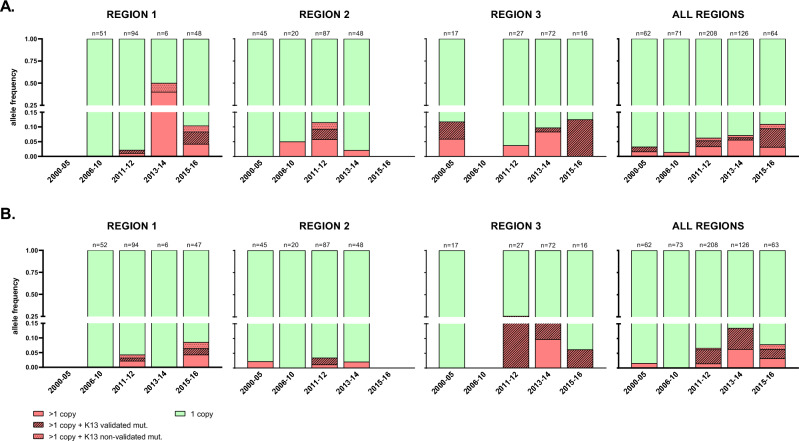
Frequency of *pm2* and *mdr1* multiple copy numbers in Vietnam (2000–2016). Bar charts indicate the percentage of samples with multiple copies (red) or single copies (green) of *pm2* (marker of piperaquine resistance; **A**) and *mdr1* (marker of mefloquine resistance; **B**). Samples with multiple copies also carrying K13 mutations are indicated with different fill patterns. Data is shown by region and years. Note that the y axis in both (**A,B**) is split in two segments (from 0 to 0.15 and from 0.25 to 1) to improve visualization of low frequencies. Region 1: Quang Tri, Quang Nam, Gia Lai and Ratanakiri (Cambodia) provinces; Region 2: Khanh Hoa, Ninh Thuan, and Binh Thuan provinces: Region 3: Binh Phuoc province.

Resistance to the artemisinin partner drug PPQ was primarily assessed based on gene copy numbers of *pm2*. Overall prevalence of *pm2* multiple copies was 6% (32/531), increasing from 3.2% (2/62) in 2000–2005 to 10.9% (7/64) in 2015–2016 (p = 0–161, Fisher’s Exact; Fig. 5A). Of note, 31.2% (10/32) of all parasites with *pm2* amplifications also carried K13 ART-R validated SNP, a proportion that was notably higher in the last period (2015–2016; Fig. 5A). Three mutations in *crt* gene (T93S, H97Y and M343L)*,* which were found to emerge in GMS associated to PPQ resistant phenotypes and ART-R genetic backgrounds^10,24^, were also covered by *crt.a* and *crt.b* amplicons in the *pf*TSCA multiplex. Both T93S and H97Y were detected but in only one sample each (Region 3, year 2014) and therefore not considered for prevalence calculations.

Resistance to mefloquine was determined based on copy number of *mdr1*. Parasites with multiple *mdr1* copies were found in 6.9% (37/532) of the samples tested. The frequency was highest in Region 3/Binh Phuoc in 2011–2012 (35%) but decreased in 2015–2016 (Fig. 5B). A total of 56.8% (21/37) of samples with *mdr1* amplifications carried a K13 ART-R validated SNP. Multiple gene copies at both *pm2* and *mdr1* were found in 0.8% of the samples (4/530), all four collected after 2012. One sample carried polymorphisms for both K13 (Y511H), *pm2* and *mdr1* (Region 1, year 2015).

### Association between markers of resistance to ACT and in vivo treatment outcomes

The study included Day 0 samples (n = 118) from two clinical trials that assessed efficacy of DHA-PPQ for uncomplicated malaria, both of them conducted in Region 2 (one in Quang Nam province 2012–2013 and one in Gia Lai 2015–2016)^36,37^. The presence of mutations in markers or modulators of DHA-PPQ resistance was analyzed relative to in vivo parasite clearance and parasite positivity at Day 3 after treatment initiation. Infections with K13 non-synonymous mutations had a significantly slower PC_1/2_ (6.6 h [IQR 4.7–7.9]) as compared to K13 wild-type infections (3.8 h [3.1–4.0], p < 0.001), either for ART-R validated or non-validated mutations (p < 0.001, Mann–Whitney U test; see Supplementary Fig. S6). Prevalence of validated K13 mutants was 89% (33/37) in Day 3-positive patients and 74% in Day 3-negative (46/62; p = 0.119, see Supplementary Table S7). The sample with triple mutations in K13 (Y511H) + *pm2* + *mdr1* was part of the Gia Lai trial and showed adequate clearance of infection by Day 3 after DHA-PPQ treatment^37^.

### Genetic markers of chloroquine resistance in Vietnam (2000–2016)

The overall frequency of the chloroquine resistant *crt* haplotypes (CVIET, CVIDT or mixed CVI[E/D]T) was > 52% across all time periods, and steadily increased over time up to 90% (64/71) in 2015–2016 (p < 0.001, chi2). Like markers of resistance to ACT, frequency of the chloroquine resistant haplotypes was lowest in Region 2 (Supplementary Fig. S7).

The *mdr1* mutation Y184F was detected in 20.5% (110/536, 20.5%). The frequency of the mutant allele increased over time in Region 1 (from 13.0 to 70.6%; see Supplementary Fig. S8), whereas it remained low and stable in Region 2 (6.2–10.6%). *mdr1*-Y184F was predominantly found in chloroquine resistant CVI[E/D]T background (n = 97) as compared to parasites with CVMNK (n = 6; p < 0.001, chi2). There was no association between presence of the 184F allele and *mdr1* copy number (p = 0.925, Fisher’s exact).

### Genetic diversity and population structure

Parasite population genetics metrics were determined from the 4 microsatellite markers in *pf*TSCA and from a 101-SNP barcode. Out of the 635 samples in the study, 542 (86.8%) returned genetic data for at least one microsatellite locus, and 340 (53.5%) for ≥ 2 loci*.* SNP barcode data was obtained from 468 out of the 529 samples processed (88.5%).

#### Complexity of infection (COI)

Based on microsatellite data, 20.5% (111/542) of all samples sequenced were single clone infections, and there was no difference in their prevalence over time (range of single clone = 13.9–27.2%, p = 0.136, Chi2; see Supplementary Table S8). The highest rate of single clone infections was observed for Region 1 in 2011–2012 (35.7%; 41/115). COI estimates from the SNP barcode data indicated that overall prevalence of single clone infections was 92.5% (433/468), remaining at > 75% in all regions and time periods (Supplementary Table S8).

#### Genetic diversity and genetic differentiation

Overall genetic diversity, measured as expected heterozygosity (*He*) at four microsatellite loci (*poly-α*, ARAII, TA81 and *pk2*) and at 101 SNPs, was stable throughout the studied period (see Supplementary Fig. S9). Microsatellite ARAII had very few different alleles in the overall population, leaving only *poly-α*, TA81 and *pk2* with sufficient variability to be informative for the analysis. The lowest *He* was found for Region 1 in 2011–2012 (median He by microsatellites = 0.07, p ≤ 0.0004 pairwise comparisons using Wilcoxon rank sum test; median *He* by 101-SNP barcode = 0.08, p ≤ 0.0004, Wilcoxon rank sum test), but increased in 2015–2016. Parasites in Region 1 in 2011–2012 and 2015–2016 showed great genetic differentiation (F_ST_) both when compared to other regions and to each other (see Supplementary Fig. S10).

#### Clustering analysis

**Figure 6:**
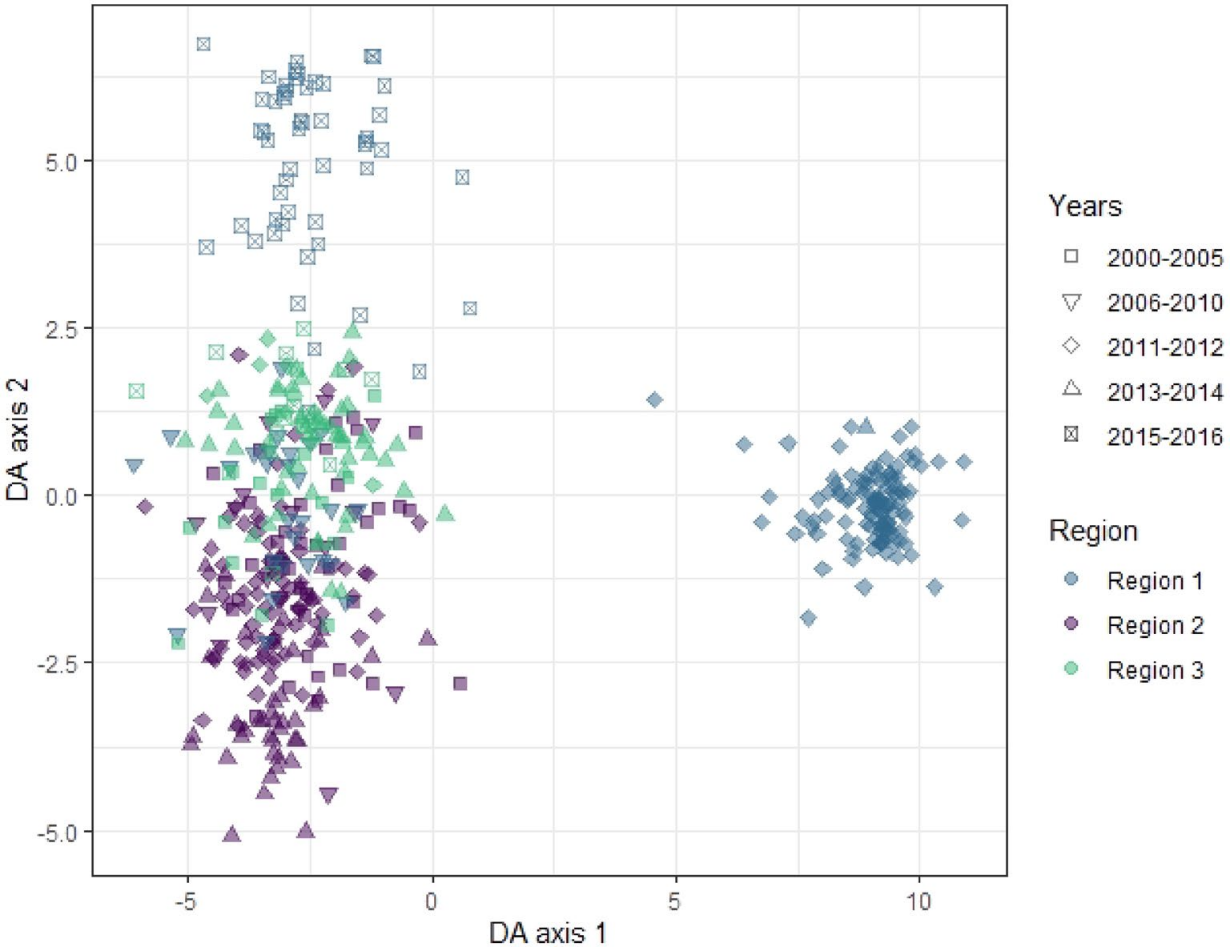
Discriminant analysis of principal component (DAPC) in samples from Vietnam (2000–2016). DAPC was performed using all markers genotyped in the *pf*TSCA and the SNP barcode. Scatter plot shows discriminant analysis (DA) eigenvalues 1 and 2, in which populations are differentiated by color (geographical region) and shape (time). Alleles contributing most to the DAPC are listed in Supplementary Table S9.

Clustering analysis was performed to explore population structure and dynamics over time. Parasites clustered predominantly by geographical region (color), rather than year (shape; see Fig. 6, Supplementary Fig. S11). The parasites from Region 1 were responsible for the majority of the variation along DA 1 and 2, with a very distinct population in 2011–2012 (blue diamonds, Fig. 6). The alleles contributing most to this separation in the DAPC were two SNPs from the barcode (Pf3D7_12_v3_1934745 and Pf3D7_04_v3_891732), followed by the K13 mutations I543T and T474I (see Supplementary Table S9). There was less distinction between samples from Region 2 and 3, as these populations overlapped.

## Discussion

In this study we developed genomic tools based on multiplex amplicon sequencing for the high-throughput molecular surveillance of antimalarial drug resistance. This tool was applied to investigate the prevalence and evolution of molecular markers of *P. falciparum* resistance to ACT in Vietnam, during the period of ART-R emergence in the GMS (2000–2016). ART-R validated mutations were detected in samples collected as early as year 2000, and steadily increased throughout the studied period, especially in Central Highlands provinces bordering Cambodia where an expansion or potential outbreak of K13-I543T was detected before the K13-C580Y mutation became predominant. Resistance to ACT partner drug PPQ remained moderately low in the surveyed period. Overall, trends in molecular markers coincided with treatment efficacy estimates from in vivo clinical trials.

The use of genomic tools to support malaria control and elimination is currently a growing area of research and is already part of NMCP malaria surveillance activities and programmatic-decision making in some countries^5,6,52,53^. Multiplex amplicon sequencing assays, such as the *pf*TSCA described here or the more recent AmpliSeq technology^7^, allow for rapid analysis of multiple markers and samples in a < 1 week-protocol with semi-automated laboratory and analysis procedures, which highly increases the potential for integration into pathogen surveillance systems of reference laboratories in endemic countries. Additional advantages of the approach are its high sensitivity and low DNA input and the combination of both drug resistance markers with direct programmatic relevance, drug resistance markers under research, and markers of population genetics to better understand dynamics and evolution of resistance. Moreover, the flexible custom designs can potentially incorporate additional targets for other surveillance purposes: *hrp2 and hrp3* genes to monitor deletions associated with false negative results in HRP2-based rapid diagnostic tests, *circumsporozoite protein* gene (*csp*) elements present in RTS,S vaccine construct, other polymorphic antigens used to characterize recurrences in therapeutic efficacy trials, or SNP barcodes with regional and country-level resolution^7^.

The analysis of a large retrospective collection of blood samples from Vietnam showed that the overall proportion of infections with K13 validated mutations was high (48.1%), with notable changes between time periods and regions surveyed. Whereas in the Central Highlands (Region 1) K13 validated mutations represented > 50% of infections since 2006, they remained a minority in coastal areas (Region 2), in agreement with the different treatment efficacy profiles reported in these two areas (see Supplementary Table S3). The type of K13 variants detected changed over time, the most notable change being that from I543T predominance to C580Y predominance in the Central Highlands between 2014 and 2015. In this region, C580Y was also reported as the most common K13 variant in the 2017–2019 period^6^. P553L was the third most frequent K13 codon change; it was detected in provinces belonging to all three regions analyzed (Gia Lai, Khanh Hoa and Binh Phuoc) in agreement with findings from previous studies^46,54^, and frequencies were stable throughout the studied years. Variant I543T was present in all regions, and its earliest detection was in 53% of the Cambodian samples collected in 2008. However, most I543T mutant parasites were from Quang Nam, likely due to an outbreak in 2011–2012, evidenced by the lowest level of genetic diversity and high genetic differentiation in Region 1 for this time period. Interestingly, another retrospective study conducted in the neighboring province of Gia Lai in the same time period did not report cases of I543T, suggesting the expansion of this variant was localized to some districts^55^.

Parasites resistant to PPQ and mefloquine, partner drugs in most of the ACT formulations used in the GMS, were uncommon, as indicated by low frequencies of copy number amplifications in *pm2* and *mdr1* genes*.* A notable exception was Binh Phuoc province in 2012, where > 20% of samples carried multiple *mdr1* copies in their genomes. The presence of CNV at these loci was frequently accompanied by presence of K13 ART-R markers, but the combination of both *pm2* and *mdr1* CNV in the same sample (i.e., PPQ + MQ resistance) was rare. Another study with samples collected immediately after 2016 reported much higher prevalence of PPQ resistant parasites^6^, what may be attributable to a rapid spread of lineages with *pm2* duplication, or to differences in genetic profiles of parasites sampled form different districts. With regards to new emerging *crt* mutations, reported by Hamilton et al*.* at frequencies of 11–21% in Vietnam in 2016–2017^10^, they were only partially targeted in our assay; mutants were rare, indicating these variants likely emerged in Vietnam after 2016.

Despite declining malaria transmission in recent years, as measured by traditional epidemiological indicators^34,56^, we did not observe a decrease in COI or genetic diversity measures during the surveyed time period. On the contrary, genetic diversity in Region 1 and 3 (derived from both SNP barcode and microsatellites) increased from 2000–2012 to 2015–2016, what may reflect evolutionary processes in the opposite direction of transmission intensity. In these areas, the frequency of parasites carrying the C580Y variant in a diverse PGB background also increased, which might suggest a population expansion after initial bottleneck events. On the other hand, genetic diversity remained stable over time in coastal provinces of Region 2, where levels of drug resistance were relatively low.

This retrospective study presents some limitations that should be considered in data interpretation. First, the convenient sampling approach included all available samples for a given province and year, resulting in unbalanced sample sizes for different temporal and geographical origins. The larger sample size for Region 1 might have resulted in higher resolution for this region as compared to others, but also allowed to establish associations between molecular and in vivo treatment efficacy data for these provinces. Future routine molecular surveillance strategies aiming at programmatic decision-making will require harmonized sampling strategies to avoid interpretation biases. Second, the molecular panel missed some *loci* of interest in the context of GMS countries, either due to incompatibility of primers in the in silico design (e.g. *exo415* and *mdr2-*T484I) or because they had not been described at the time of the assay validation (e.g. KEL1 lineage SNP or *crt* variants conferring resistance to PPQ^10^). Third, the number of microsatellite markers for genetic diversity was insufficient to uncover parasite population structuring at the geographical scale needed, although in this case resolution increased by using the 101-SNP barcode data in population genetic analysis. An additional factor limiting optimal microsatellite allele calling was the use of algorithms for tandem repeat calling in NGS data. These algorithms are generally designed for diploid organisms and not samples of unknown ploidy (as it is often encountered in malaria complex infections), and require high and balanced allele depth, which resulted in missing microsatellite alleles in the present dataset. Our team has currently transitioned to AmpliSeq® technology (Illumina) and added SNP barcodes in panels applied to recent surveillance studies^7,57^.

In conclusion, multiplexed targeted amplicon sequencing combined with adequate sampling strategies are a powerful tool for routine malaria molecular surveillance. The application of *pf*TSCA assay to a retrospective collection of samples from Vietnam allowed to characterize in detail the increase in *P. falciparum* ART-R observed in the past two decades, and to detect regional and temporal changes in the parasite population. Population genetic analysis requires higher resolution markers like SNP-barcodes and cannot rely only on a reduced number of microsatellites.

### Supplementary Information

Supplementary Information 1.

Supplementary Information 2.

Supplementary Information 3.

## Data Availability

The datasets (fastq files) generated and/or analyzed during the current study are available in the NCBI Sequence Read Archive (SRA) repository under BioProject accession number PRJNA957102, and individual library accession numbers are listed in the Supplementary Data S1 file. Variant call format (vcf) files are available from the corresponding author upon request.
