# Author Correction: Molecular surveillance of *Plasmodium falciparum* drug-resistance markers in Vietnam using multiplex amplicon sequencing (2000–2016)

**DOI:** 10.1038/s41598-023-43996-w

**Published:** 2023-10-11

**Authors:** Eduard Rovira-Vallbona, Johanna Helena Kattenberg, Nguyen Van Hong, Pieter Guetens, Hideo Imamura, Pieter Monsieurs, Driss Chiheb, Annette Erhart, Bui Quang Phuc, Nguyen Xuan Xa, Anna Rosanas-Urgell

**Affiliations:** 1 Department of Biomedical Sciences, Institute of Tropical Medicine, 2000 Antwerp, Belgium; 2 National Institute of Malariology, Parasitology and Entomology, Hanoi, 10200 Vietnam; 3 Present Address: ISGlobal, Hospital Clínic/Universitat de Barcelona, 08036 Barcelona, Catalonia Spain; 4 Present Address: Vrije Universiteit Brussel, Campus Jette, 1090 Brussels, Belgium; 5 Present Address: UZ Brussel, Centre for Medical Genetics, 1090 Brussels, Belgium; 6 Present Address: Medical Research Council Unit, The Gambia at the London School of Hygiene and Tropical Medicine, Fajara, The Gambia

Correction to: *Scientific Reports* https://doi.org/10.1038/s41598-023-40935-7, published online 25 August 2023

The original version of this Article contained an error in the Data availability section, where the SRA Bioproject accession number for DNA sequences was incorrect.

“The datasets (fastq files) generated and/or analyzed during the current study are available in the NCBI Sequence Read Archive (SRA) repository under BioProject accession number PRJNA855317, and individual library accession numbers are listed in the Supplementary Data S1 file. Variant call format (vcf) files are available from the corresponding author upon request.”

now reads:

"The datasets (fastq files) generated and/or analyzed during the current study are available in the NCBI Sequence Read Archive (SRA) repository under BioProject accession number PRJNA957102, and individual library accession numbers are listed in the Supplementary Data S1 file. Variant call format (vcf) files are available from the corresponding author upon request."

The original Article has been corrected.

