# Supplementary material for: Molecular surveillance of Plasmodium falciparum drug-resistance markers in Vietnam using multiplex amplicon sequencing (2000–2016)
Source: Sci Rep. 2023 Aug 25;13:13948. doi: 10.1038/s41598-023-40935-7 (PMC10457381; doi:10.1038/s41598-023-40935-7)
Supplement: Supplementary file 1 — Supplementary Information 1. [file 41598_2023_40935_MOESM1_ESM.pdf]

Molecular surveillance of *Plasmodium falciparum* drug-resistance markers in Vietnam using multiplex amplicon sequencing (2000-2016)

Supplementary Figures and Tables

Contents

Supplementary Figures.. 3

Supplementary Figure S1. Sample selection and sample processing flow-chart........ 3

Supplementary Figure S2. Coverage of *kelch13* gene sequence in *pf*TSCA assay...... 4

Supplementary Figure S3. Detectable frequency of minor clones using parasite mixes... 5

Supplementary Figure S4. Performance of the *pf*TSCA assay by parasite density..... 6

Supplementary Figure S5. Frequencies of *arps10*-V127M, *fd*-D193Y and *crt*-N326S individual alleles in Vietnam (2000-2016). 7

Supplementary Figure S6. K13 mutations and parasite clearance half-life after dihydroartemisinin-piperaquine (DHA-PPQ) treatment... 8

Supplementary Figure S7. Frequency of crt 72-76 haplotype variants as marker of chloroquine resistance in Vietnam (2000-2016)... 9

Supplementary Figure S8. Frequency of *mdr1*-Y184F allele in Vietnam (2000-2016)... 10

Supplementary Figure S9. Expected Heterozygosity (He) in Vietnam (2000-2016). 11

Supplementary Figure S10. Genetic differentiation (F_{ST}) between *P. falciparum* populations in Vietnam (2000-2016)... 12

Supplementary Figure S11. Principal component analysis (PCA) in study samples from Vietnam (2000-2016)... 13

Supplementary Tables.. 14

Supplementary Table S1. Design of the multiplex *pf*TSCA assay and oligonucleotide probes used... 14

Supplementary Table S2. Read counts in the multiplex *pf*TSCA assay for positive and negative controls.. 15

Supplementary Table S3. Clinical resistance to dihydroartemisinin-piperaquine (DHA-PPQ) reported in Vietnam provinces... 16

Supplementary Table S4. Read counts in the multiplex *pf*TSCA assay for study samples (n=635)... 18

Supplementary Table S5. Genotyping of study samples by MalariaGEN SpotMalaria ……… 19
Supplementary Table S6. K13 mutations identified in study samples from Vietnam (2000-2016)……………………………………………………………………………………………… 20
Supplementary Table S7. Drug resistance markers and Day 3 positivity rate after dihydroartemisinin-piperaquine (DHA-PPQ) treatment………………………………………. 21
Supplementary Table S8. Complexity of infection (COI) in study samples from Vietnam (2000-2016)………………………………………………………………………………………………. 22
Supplementary Table S9. Discriminant Analysis of Principal Components (DAPC) loadings……………………………………………………………………………………………………. 23

Supplementary Figures

Supplementary Figure S1. Sample selection and sample processing flow chart. DBS, dried blood spots; CNV, copy number variation; sWGA, selective whole genome amplification.

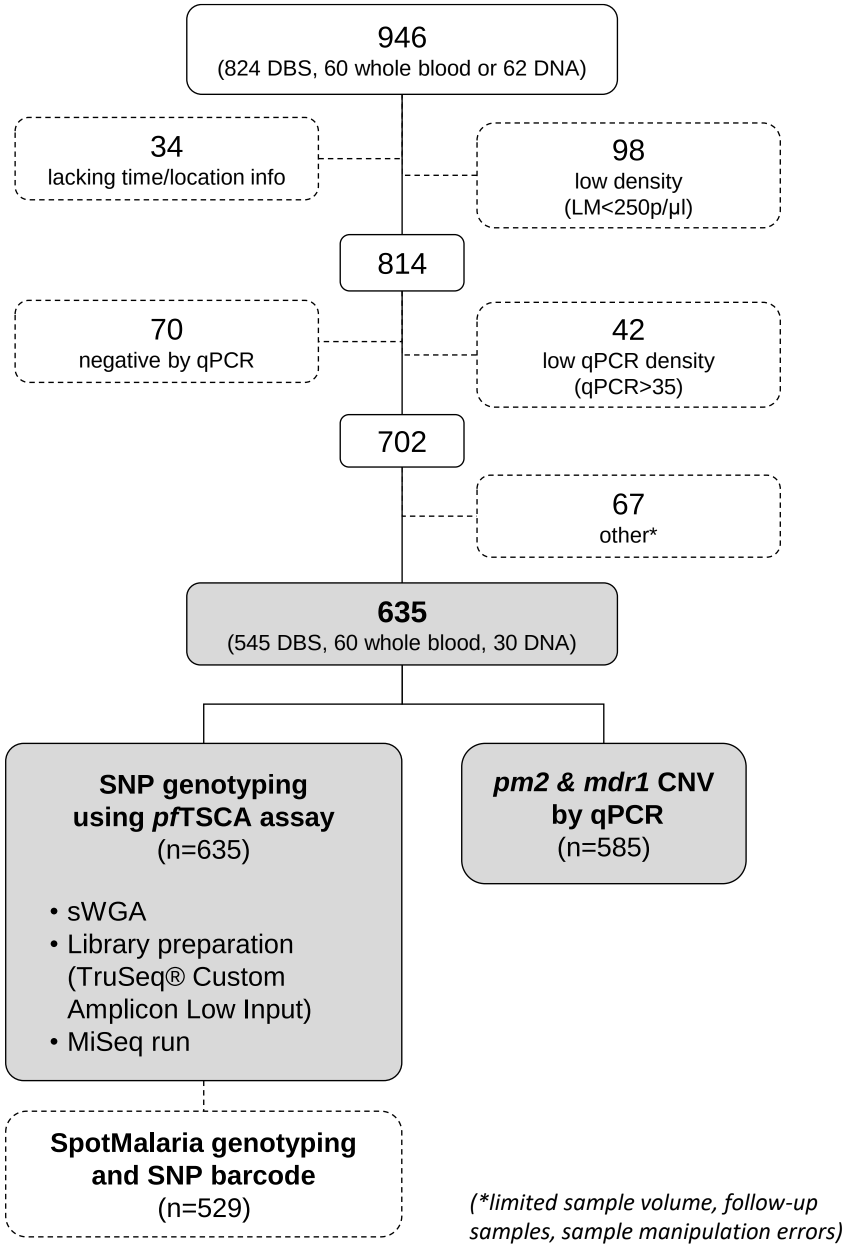

Supplementary Figure S2. Coverage of *kelch13* (K13) gene sequence in *pf*TSCA assay. Each black line represents an amplicon of length ranging 226 to 273 bp.

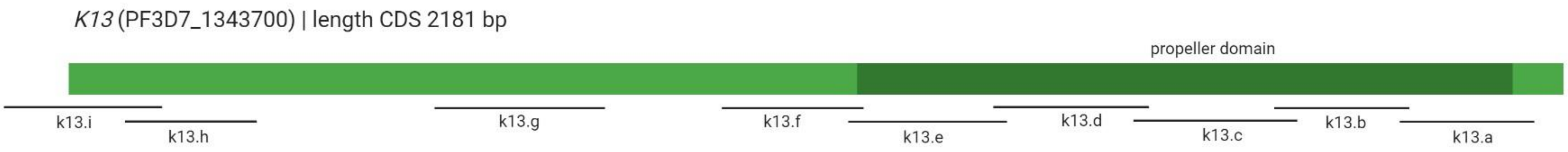

Supplementary Figure S3. Detectable frequency of minor clones using parasite mixes. DNA from *P.falciparum* strains 3D7 (reference WT strain), CamWT_C580Y (K13-C580Y, *arps10*-V127M) and Dd2 (*crt*-CVIET haplotype and *fd*-D193Y) were mixed at the following ratios: a) 3D7:Cam1251 at 90:10, 95:5 and 99:1; b) 3D7:Dd2 at 50:50, 80:20, 90:10, 95:5 and 99:1; c) Dd2:Cam1251 at 90:10. Bars show the observed ratio of WT (dark or light grey) *versus* mutant allele (colored) for each mix in the TSCA assay.

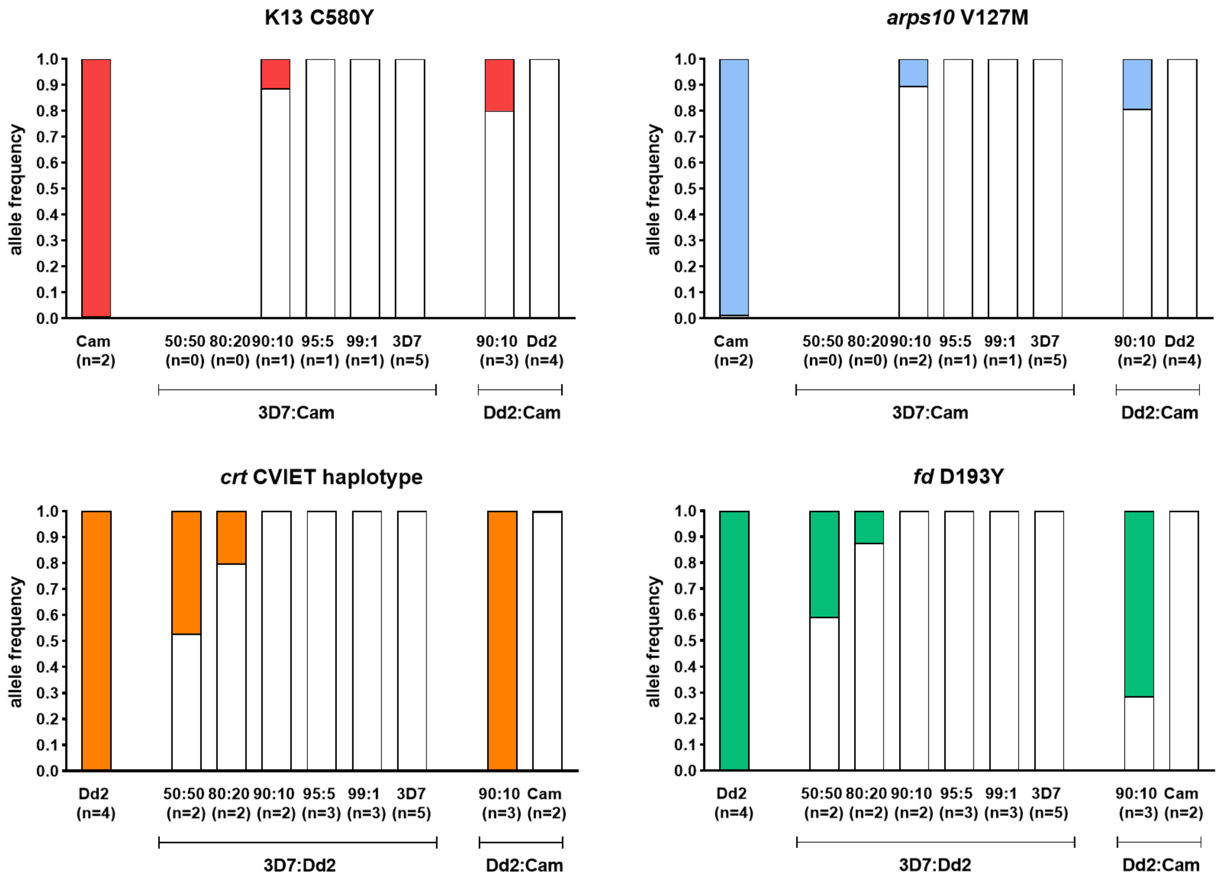

Supplementary Figure S4. Performance of the *pf*TSCA assay by parasite density. Plots show the number of amplicons above the read-depth cut-off for each sample (x axis) by parasite density (y axis): A, parasite density as parasites/µl measured by microscopy, all samples; B, parasite density measured by microscopy, dried blood spot samples only; C, parasite density measured by microscopy, whole blood EDTA samples only; D, parasite density quantified by *var*ATS qPCR (Ct value) .

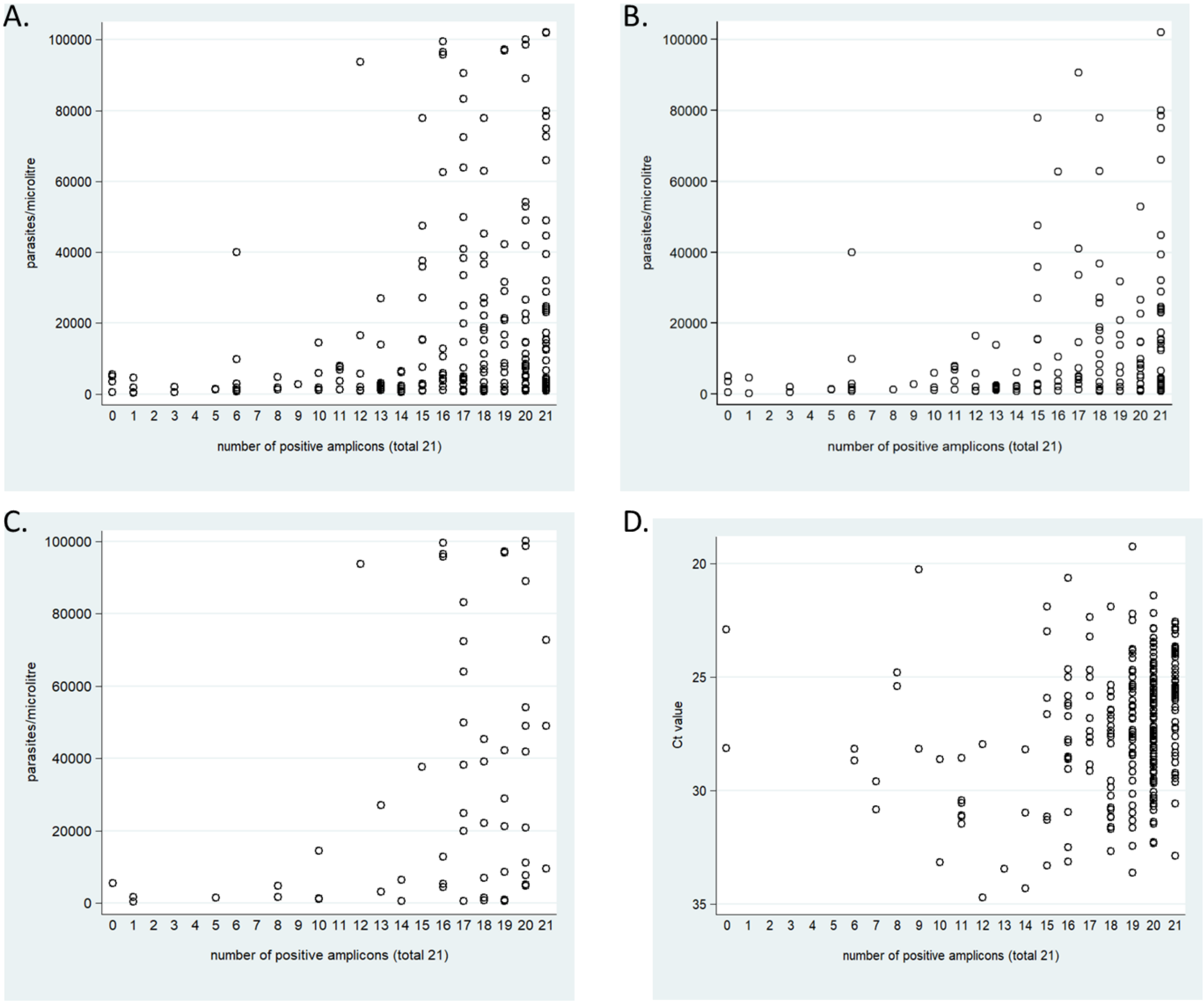

Supplementary Figure S5. Frequencies of *arps10-V127M, fd-D193Y and crt-N326S* individual alleles in Vietnam (2000-2016). Bar charts indicate the percentage of samples with mutant (red) or wild type alleles (green) for either *arps10-V127M* (panel A; N=606)*, fd-D193Y* (panel B; N=550) and *crt-N326S* (panel C; N=427). Data is shown by region and years. Region 1: Quang Tri, Quang Nam, Gia Lai and Ratanakiri (Cambodia) provinces; Region 2: Khanh Hoa, Ninh Thuan, and Binh Thuan provinces: Region 3: Binh Phuoc province.

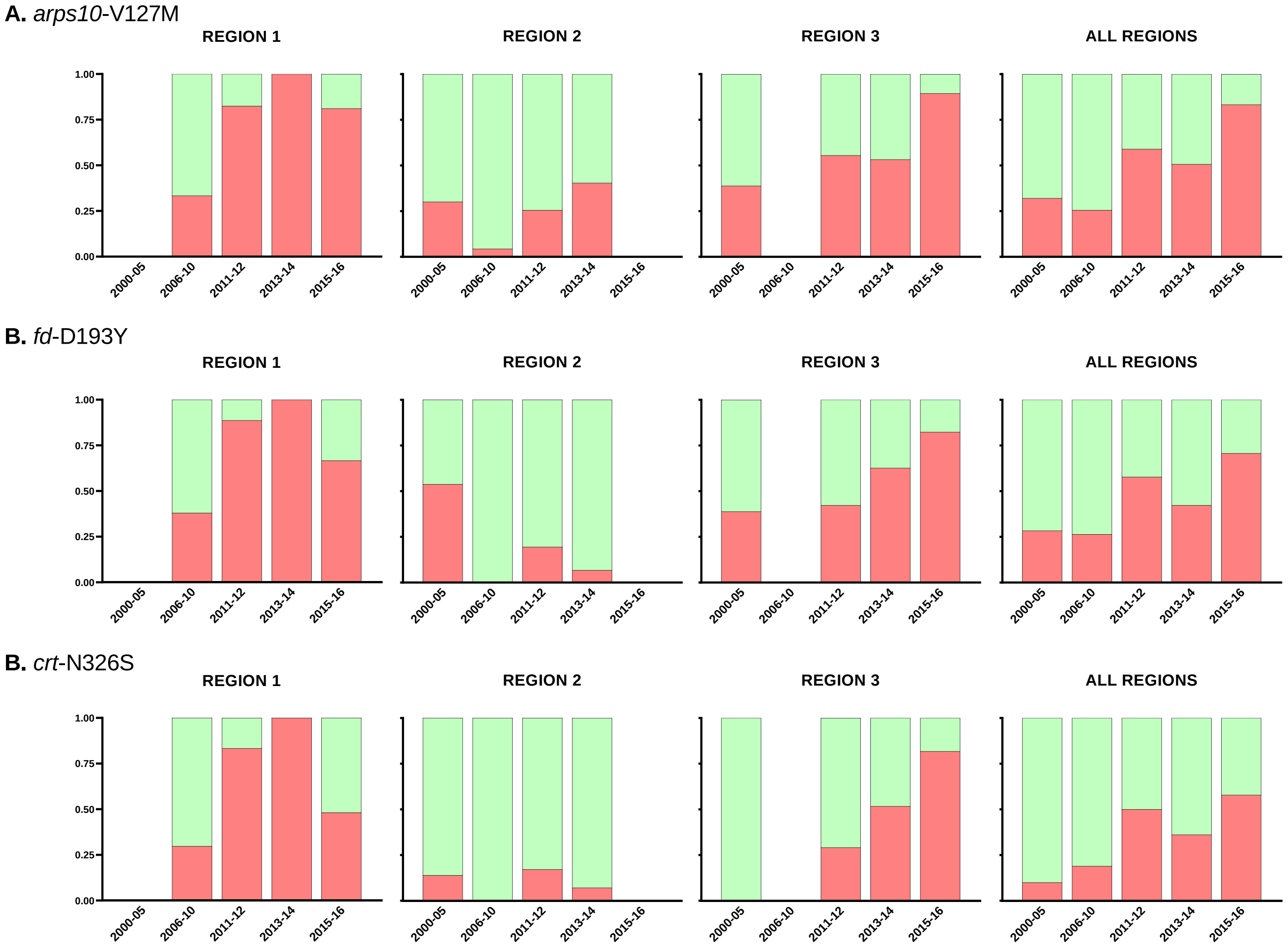

Supplementary Figure S6. K13 mutations and parasite clearance half-life after dihydroartemisinin-piperaquine (DHA-PPQ) treatment. Parasite clearance half-life after DHA-PPQ administration stratified by K13 genotype (N=94). Non-validated K13 mutations are shown in white circles. Validated mutations (*i.e*. those with a confirmed association with ART-R) are shown in dark grey circles. WT, wild type.

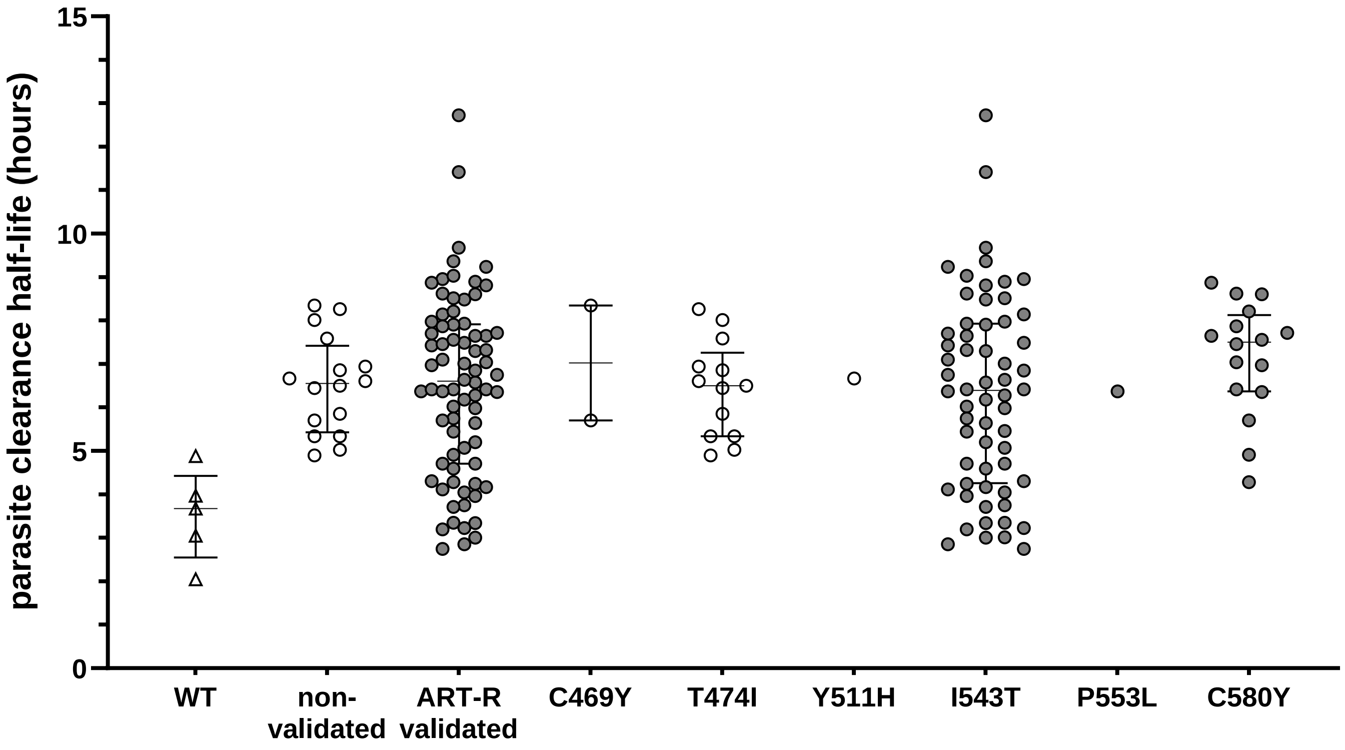

Supplementary Figure S7. Frequency of *crt* 72-76 haplotype variants as marker of chloroquine resistance in Vietnam (2000-2016). Bar charts indicate the percentage of samples with mutant (red) or wild type haplotypes (green). Specific haplotypes are indicated with different fill patterns. Data is shown by region and years. Total sample size was N=544. Region 1: Quang Tri, Quang Nam, Gia Lai and Ratanakiri (Cambodia) provinces; Region 2: Khanh Hoa, Ninh Thuan, and Binh Thuan provinces: Region 3: Binh Phuoc province.

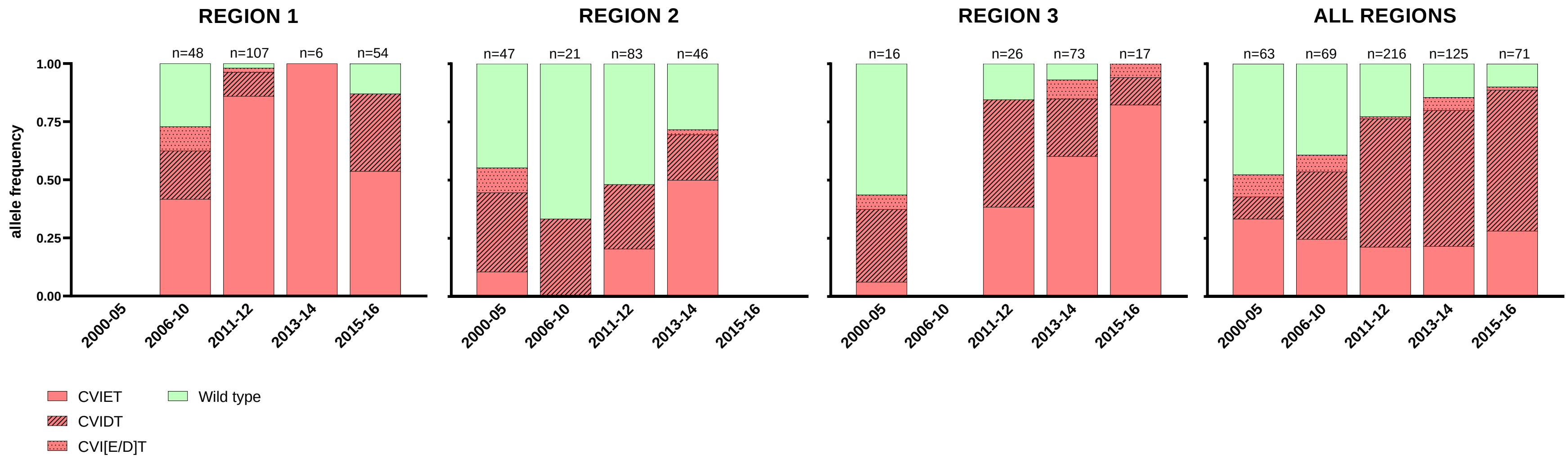

Supplementary Figure S8. Frequency of *mdr1*-Y184F allele in Vietnam (2000-2016). Bar charts indicate the percentage of samples with mutant (red) or wild type alleles (green). Data is shown by region and years. The total sample size was N=536. Region 1: Quang Tri, Quang Nam, Gia Lai and Ratanakiri (Cambodia) provinces; Region 2: Khanh Hoa, Ninh Thuan, and Binh Thuan provinces: Region 3: Binh Phuoc province.

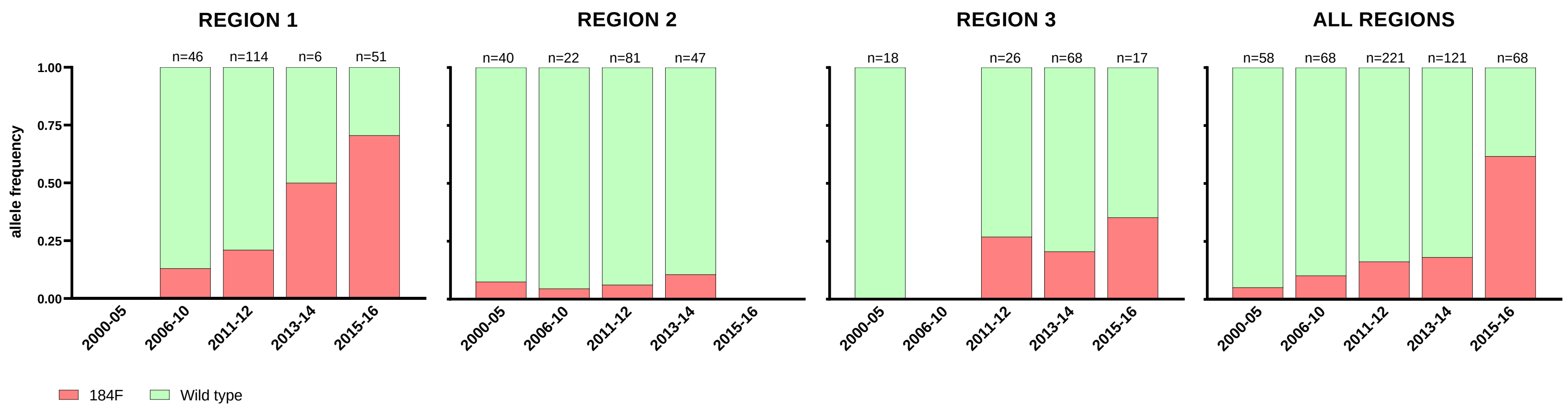

Supplementary Figure S9. Expected Heterozygosity (*He*) in Vietnam (2000-2016). A) *He* for microsatellite markers (*poly-α*, ARAII, TA81 and *pk2*), by region and time. B) *He* for the 101 SNPs barcode, by region and time.

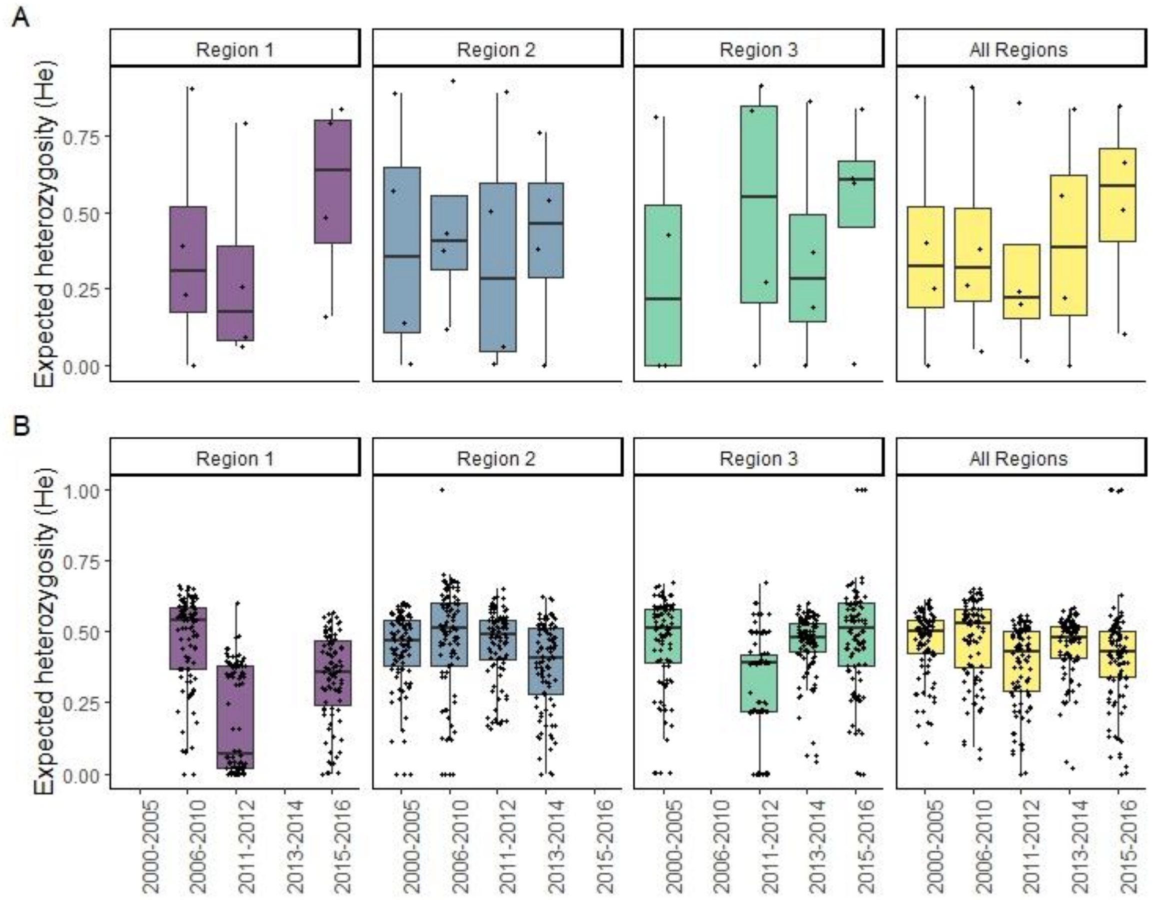

Supplementary Figure S10. Genetic differentiation (F_{ST}) between *P. falciparum* populations in Vietnam (2000-2016). A) F_{ST} using microsatellite loci (*poly-α*, ARAII, TA81 and *pk2*) by region and time. B) F_{ST} using 101 SNPs barcode loci by region and time.

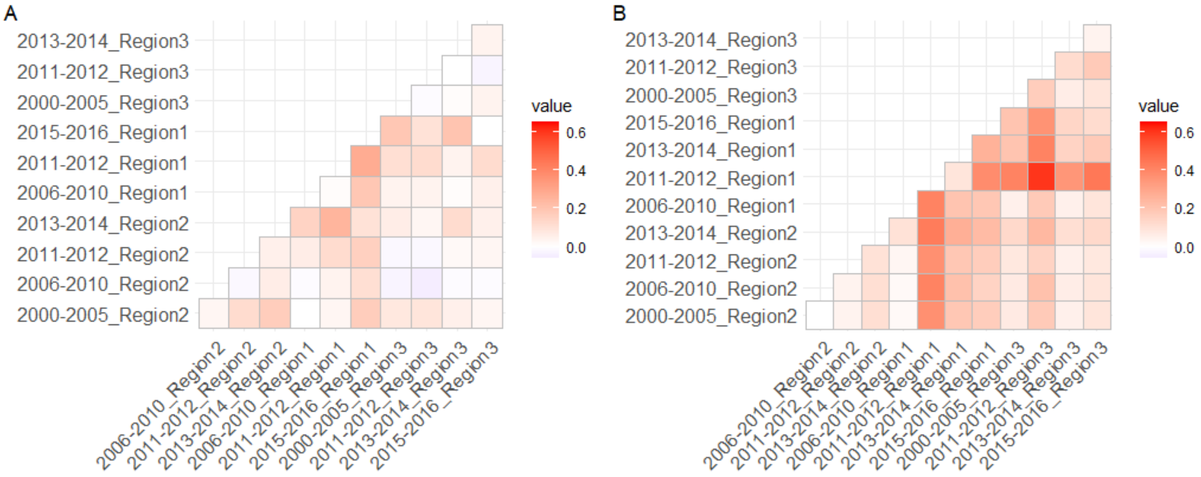

Supplementary Figure S11. Principal component analysis (PCA) in study samples from Vietnam (2000-2016). PCA was conducted using all markers in *pf*TSCA (microsatellites *poly-α*, ARAII, TA81, *pk2* and drug resistance markers) and the 101 SNPs barcode. PCA is shown along the first two principal components. Samples are colored by geographical region, and shapes indicate the different periods of collection in years. Samples from Region 1 and years 2011-2012 (blue diamonds) are responsible for the largest variation and cluster separately.

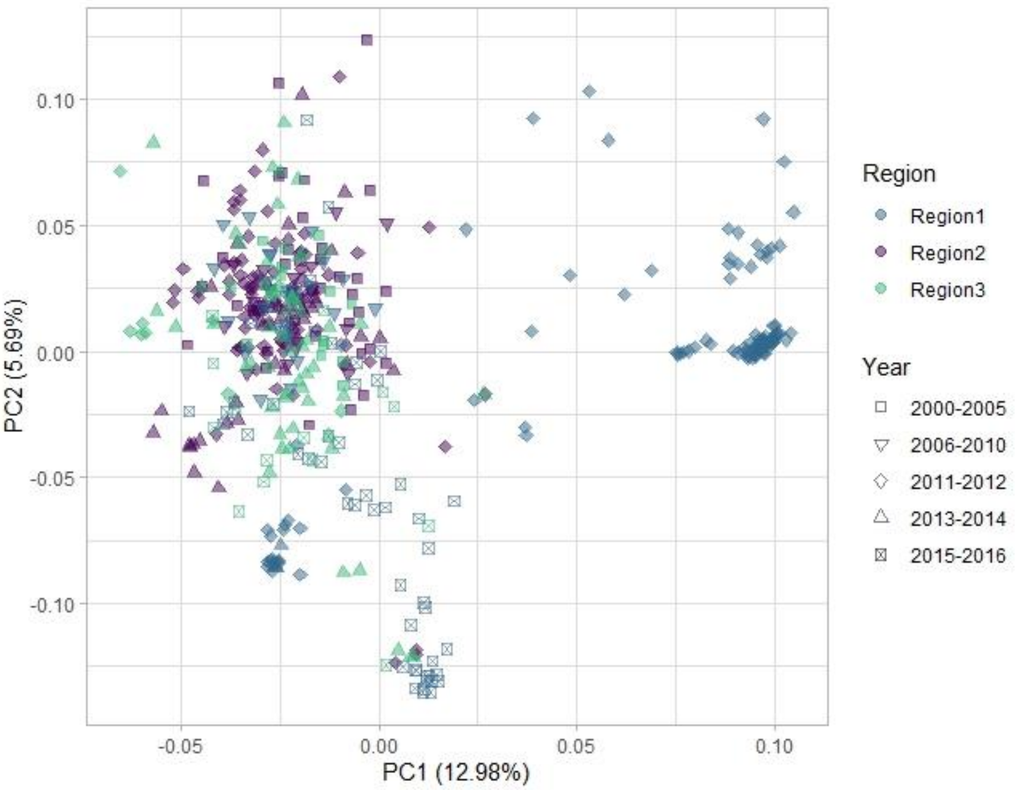

Supplementary Tables

Supplementary Table S1. Design of the multiplex *pf*TSCA assay and oligonucleotide probes used.

gene	target	% target sequence covered	custom probes sequence and genomic location							
			amplicon name	chromosome	start position	end position	probe strand	upstream locus-specific oligo	upstream locus-specific oligo	size (bp)
**Markers of ART partial resistance:**										
Kelch13 (PF3D7_1343700)	codon 1-440	73%	k13.i	Pf3D7_13_v3	1724894	1725166	-	CTGGTGAAAAGAAATGACATGAATTTAGA	CTAATAAGGCATATGGAAATTGTTCCC	273
			k13.h	Pf3D7_13_v3	1725112	1725370	+	TTGGGGGATATGATGGCTCTTCTATTA	AGCTAGAAGTTCAGGAGCAGCTTTTAA	259
			k13.g	Pf3D7_13_v3	1725316	1725588	-	CGATGATCATATGCTTCTACATTCGGT	TTGGTAGACATAGGTGTACACATACGC	273
	propeller domain	100%	k13.f	Pf3D7_13_v3	1725534	1725761	+	CCGTTAACTATACCCATACCAAAAGATT	AAAGCTTATTTTGGAAGTGCTGTATTG	228
			k13.e	Pf3D7_13_v3	1725706	1725932	-	TCTTTCAACAAGGCTTCACTTTCACTT	AGATGTTTCAAAAATAGCTCCACCAAC	227
			k13.d	Pf3D7_13_v3	1725876	1726112	+	GAAGAACATAGGAAACGATTTGATGAA	GACATACCTTAACACAACAAAAAGATTCA	237
			k13.c	Pf3D7_13_v3	1726214	1726478	+	GCAGCAAATCTTATAAATGATGATTCTGG	TGGAAAGAGTACGATTGTACAAAGAAT	265
			k13.b	Pf3D7_13_v3	1726754	1726979	-	TCATATCAATGGATTCTAATAGGCTATCTT	AAAATTCGAGATACTATTTGCTTTTGTTTT	226
			k13.a	Pf3D7_13_v3	1726922	1727148	+	AGGCGTAAATATTCGTGTTATAATTTCTC	CTATGACGTATGATAGGGAATCTGGTG	227
**Modulators of ART-R / ART-R genetic background:**										
arps10 (PF3D7_1460900)	V127M	100%	arps10	Pf3D7_14_v3	2481043	2481307	+	AACCCACAAATCTGGGTAATTTCACTG	TGGGGAGATCGCAAAAAGGTAAATCT	265
fd (PF3D7_1318100)	D193Y	100%	fd	Pf3D7_13_v3	748269	748543	+	CGCAGCAAAATTAGTCGAAGGAGAAGT	TTTGGTATGGATATGATTGAAATGGGG	275
crt (PF3D7_0709000)	N326S	100%	crt.b	Pf3D7_07_v3	405332	405604	+	TGCTATTGCTGGACCTTGTATACAACT	AGAAGGAAAACAATGCGAAGGTTTTCT	273
MAL10:688956	MAL10:688956	100%	MAL10	Pf3D7_10_v3	688872	689138	+	ATGGAAACATTTATGCCATCAACATTT	TTCATATATTAATTCGCCCATTCATTACA	267
RAD5-hom. (PF3D7_1343400)	S1158A (MAL13)	100%	MAL13	Pf3D7_13_v3	1718263	1718507	+	TGAGTTAAATGGAAATAATCGTCTGCA	CTCATTGGAATGATCTACTTTACTAAAACT	245
**Markers of resistance to other antimalarial drugs:**										
crt (PF3D7_0709000)	72-76_CVI[E/D]T	100%	crt.a	Pf3D7_07_v3	403525	403756	+	TTGAATTTCCCTTTTTATTTCCAAATAAGG	AAAATAAGTTTAAACACATGAGCACATTT	232
mdr1 (PF3D7_0523000)	Y184F	100%	mdr1	Pf3D7_05_v3	958410	958638	+	TCTCCACAATAACTTGCAACAGTTCTT	AACCTAAAAAGGAACTGGCATATGTAA	229
**Markers of population structure:**										
poly-α (PF3D7_0411900)	(AAT)11-20	100%	MS_poly-α	Pf3D7_04_v3	532155	532407	+	TTGATTGTATTGTGGAAGATAAAAAGAGT	GACGATGGACCCAAAAAGATCGAACA	253
ARAII (PF3D7_1110400)	(AAT)9-12	100%	MS_ARAII	Pf3D7_11_v3	416186	416426	+	TTTTGGTCAAGTGGTACAGATCTTTTT	GGTGATTCATATGTACATTTGAACCTC	241
TA81 (PF3D7_0529800)	(AAT)7-15	100%	MS_TA81.a	Pf3D7_05_v3	1214217	1214449	+	TCATACATTTCACACAACACAGGATTA	TTGCTGTAATTCTTCATACTTGTCTGA	233
			MS_TA81.b	Pf3D7_05_v3	1214393	1214648	-	AAGGAATGATAACATTAATGGTGATGGT	TGTGATACACATGATTCAAATTATAATCGT	256
pk2 (PF3D7_1238900)	(TTA)8-13	89%	MS_pk2	Pf3D7_12_v3	1611177	1611401	+	AATAGTTATTCCTTTCATCGATACTACGA	AGCATTATTCGGATTATGTTCTTTATGAA	225

Supplementary Table S2. Read counts in the *pf*TSCA assay for positive and negative controls.

gene	target	amplicon name	negative controls (n=12)							positive controls (n=33)						
			controls with reads	read count						controls above cut-off		read count				
				average	SD	median	p25	p75	max	n	%	average	SD	median	p25	p75
**Markers of ART partial resistance:**																
Kelch13 (PF3D7_1343700)	codon 1-440	k13.i	1	0	0	0	0	0	1	9	27,3%	117	141	53	22	117
		k13.h	12	12	9	8	4	20	27	33	100,0%	15063	8281	12385	9313	18830
		k13.g	2	1	1	0	0	0	4	33	100,0%	555	405	470	234	776
	propeller domain	k13.f	7	2	3	2	0	2	9	33	100,0%	4881	3389	5035	1572	7526
		k13.e	9	6	7	4	1	10	20	33	100,0%	21584	16794	14519	7760	30066
		k13.d	11	4	5	2	1	5	16	33	100,0%	9908	5517	9802	6569	13766
		k13.c	9	6	6	4	1	10	18	32	97,0%	14830	10827	15242	6938	22848
		k13.b	10	4	4	3	1	5	13	33	100,0%	17918	11915	14464	8207	25640
		k13.a	9	4	4	3	1	7	15	33	100,0%	13096	7489	13505	9222	17723
**Modulators of ART-R / ART-R genetic background:**																
arps10 (PF3D7_1460900)	V127M	arps10	12	25	19	23	6	42	55	33	100,0%	33801	28450	27805	19451	43703
fd (PF3D7_1318100)	D193Y	fd	9	2	2	1	1	3	5	30	90,9%	5022	4514	3993	1263	5995
crt (PF3D7_0709000)	N326S	crt.b	1	0	1	0	0	0	2	20	60,6%	62	69	36	28	70
MAL10:688956	MAL10:688956	MAL10	0	0	0	0	0	0	0	16	48,5%	297	279	304	26	458
RAD5-homol. (PF3D7_1343400)	S1158A (MAL13)	MAL13	6	2	4	1	0	3	14	33	100,0%	6993	4828	7074	2333	10060
**Markers of resistance to other antimalarial drugs:**																
crt (PF3D7_0709000)	72-76_CVI[E/D]T	crt.a	7	2	3	2	0	3	9	33	100,0%	1653	1542	1304	236	2577
mdr1 (PF3D7_0523000)	Y184F	mdr1	3	1	1	0	0	1	3	33	100,0%	2868	1784	2452	1777	4104
**Markers of population structure:**																
poly-α (PF3D7_0411900)	(AAT)11-20	MS_poly-a	1	0	1	0	0	0	3	30	90,9%	789	1022	521	128	979
ARAII (PF3D7_1110400)	(AAT)9-12	MS_ARAII	12	45	47	34	3	74	132	33	100,0%	24839	16457	22805	11787	37240
TA81 (PF3D7_0529800)	(AAT)7-15	MS_TA81.a	2	0	1	0	0	0	2	27	81,8%	437	339	527	96	706
		MS_TA81.b	11	15	16	10	3	24	44	33	100,0%	10343	6102	10573	4525	14651
pk2 (PF3D7_1238900)	(TTA)8-13	MS_pk2	6	3	6	1	0	4	18	31	93,9%	2251	2339	1475	311	3367

Supplementary Table S3. Clinical resistance to dihydroartemisinin-piperaquine (DHA-PPQ) reported in Vietnam provinces. Treatment failure rate and parasite positivity rate at day 3 after treatment initiation, reported by therapeutic efficacy studies (TES) conducted in different Vietnam provinces. Note that communes and districts where reported TES were conducted may not be the same as those sampled in the present study.

Geographical regions in data analysis	Administrative divisions		Indicators of *in vivo* efficacy of DHA-PPQ	
	Province	Region	Treatment failure (%)[1]	Day 3 positivity (%)
Region1	Quang Tri	North Central Coast	0% (2015) [2]	27% (2015) [2]
	Quang Nam	South Central Coast	<10% (2019) [3]	29% (2013) [3]
	Gia Lai	Central Highlands	0% (2015) - 40% (2019) [4]	39% (2016) [4]
	Ratanakiri (Cambodia)	*NA*	26% (2019) [5]	7%(2013) - 51% (2018) [5,6]
Region2	Khanh Hoa	South Central Coast	<10% (2014) [7]	>10% (2014) [7]
	Ninh Thuan	South Central Coast	<10% (2015) [7,8]	0-32% (2015) [2,8,9]
	Binh Thuan	South Central Coast	No TF reported (NIMPE)	-
Region3	Binh Phuoc	Southeast	>10% (2015) - >50% (2018) [5,7,10]	57% (2015) - 75% (2018) [5,6,8]

NA: not applicable; TF: treatment failure; NIMPE: National Institute of Malariology, Entomology and Parasitology (Ministry of Health).

References in Table S1:

1. World Health Organization. Methods for surveillance of antimalarial drug efficacy. https://www.who.int/publications/i/item/9789241597531 (2009)
2. Pau, M. C. *et al.* Clinical impact of the two ART resistance markers, K13 gene mutations and DPC3 in Vietnam. *PLoS One* **14**:e0214667; 10.1371/journal.pone.0214667 (2019).
3. Thriemer, K. *et al.* Delayed parasite clearance after treatment with dihydroartemisinin-piperaquine in Plasmodium falciparum malaria patients in central Vietnam. *Antimicrob Agents Chemother* **58**, 7049–7055 (2014).
4. Rovira-Vallbona, E. *et al.* Efficacy of dihydroartemisinin/piperaquine and artesunate monotherapy for the treatment of uncomplicated Plasmodium falciparum malaria in Central Vietnam. *J Antimicrob Chemother* **75**, 2272–2281 (2020).
5. van der Pluijm, R. W. *et al.* Determinants of dihydroartemisinin-piperaquine treatment failure in Plasmodium falciparum malaria in Cambodia, Thailand, and Vietnam: a prospective clinical, pharmacological, and genetic study. *Lancet Infect Dis* **19**, 952–961 (2019).
6. Ashley, E. A. *et al.* Spread of artemisinin resistance in Plasmodium falciparum malaria. *N Engl J Med* **371**, 411–423 (2014).
7. World Health Organization. Artemisinin resistance and artemisinin-based combination therapy efficacy. Status report. https://apps.who.int/iris/handle/10665/274362 (2018)

8. Thanh, N. V. *et al.* Rapid decline in the susceptibility of Plasmodium falciparum to dihydroartemisinin-piperaquine in the south of Vietnam. *Malar J* **16**, 27 (2017).
9. Phong, N. C. *et al.* Susceptibility of Plasmodium falciparum to artemisinins and Plasmodium vivax to chloroquine in Phuoc Chien Commune, Ninh Thuan Province, south-central Vietnam. *Malar J* **18**, 10 (2019).
10. Phuc, B. Q. *et al.* Treatment Failure of Dihydroartemisinin/Piperaquine for Plasmodium falciparum Malaria, Vietnam. *Emerg Infect Dis* **23**, 715–717 (2017).

Supplementary Table S4. Read counts in the multiplex *pf*TSCA assay for study samples. N=635

gene	target	amplicon	samples above cut-off		read depth				
			n	%	average	SD	median	p25	p75
**Markers of ART partial resistance:**									
Kelch13 (PF3D7_1343700)	codon 1-440	k13.i	321	50,6%	341	1227	85	27	242
		k13.h	623	98,1%	11966	20696	7475	2942	14065
		k13.g	514	80,9%	420	828	205	80	442
	propeller domain	k13.f	585	92,1%	2700	5916	1217	337	3285
		k13.e	593	93,4%	4434	6998	2647	621	5865
		k13.d	588	92,6%	4304	8749	2270	580	5612
		k13.c	574	90,4%	5192	10181	2826	633	6769
		k13.b	572	90,1%	4021	7731	2321	442	5334
		k13.a	577	90,9%	4684	9037	2429	560	6228
**Modulators of ART-R / ART-R genetic background:**									
arps10 (PF3D7_1460900)	V127M	arps10	617	97,2%	26303	45580	16406	4718	32865
fd (PF3D7_1318100)	D193Y	fd	561	88,3%	2980	7959	1082	222	3017
crt (PF3D7_0709000)	N326S	crt.b	436	68,7%	509	1742	142	48	411
MAL10:688956	MAL10:688956	MAL10	218	34,3%	83	158	44	21	83
RAD5-hom. (PF3D7_1343400)	S1158A (MAL13)	MAL13	526	82,8%	1608	3664	494	129	1556
**Markers of resistance to other antimalarial drugs:**									
crt (PF3D7_0709000)	72-76_CVI[E/D]T	crt.a	558	87,9%	3160	14465	941	313	2520
mdr1 (PF3D7_0523000)	Y184F	mdr1	547	86,1%	1391	4450	595	224	1270
**Markers of population structure:**									
poly-α (PF3D7_0411900)	(AAT)11-20	MS_poly-α	497	78,3%	555	1782	147	41	427
ARAII (PF3D7_1110400)	(AAT)9-12	MS_ARAII	617	97,2%	61570	78457	36281	9288	93452
TA81 (PF3D7_0529800)	(AAT)7-15	MS_TA81.a	526	82,8%	585	1611	248	98	562
		MS_TA81.b	612	96,4%	14416	29924	6724	2323	15274
pk2 (PF3D7_1238900)	(TTA)8-13	MS_pk2	584	92,0%	5838	20498	1561	432	5284

Supplementary Table S5. Genotyping of study samples by MalariaGEN SpotMalaria. Tables shows agreement between Sanger sequencing or MassArray and *pf*TSCA results. Genotypes were stratified based on their coincidence with either the major or the minor allele in *pf*TSCA assay.

gene	target	N	coincident genotype, n (%)		discordant genotype, n (%)
			with *pf*TSCA major allele	with *pf*TSCA minor allele	
kelch13 (PF3D7_1343700)	propeller domain	68	64 (94.1%)	1 (1.5%)	3 (4.4%)
arps10 (PF3D7_1460900)	V127M	415	393 (94.7%)	16 (3.9%)	6 (1.4%)
fd (PF3D7_1318100)	D193Y	410	401 (97.8%)	6 (1.5%)	3 (0.7%)
crt (PF3D7_0709000)	N326S	332	327 (98.5%)	2 (0.6%)	3 (0.9%)
crt (PF3D7_0709000)	CVI[E/D]T	401	386 (96.3%)	9 (2.2%)	6 (1.5%)
mdr1 (PF3D7_0523000)	Y184F	394	386 (98.0%)	1 (0.2%)	7 (1.8%)
Total		1626	1957 (96.9%)	35 (1.7%)	28 (1.4%)

Supplementary Table S6. K13 mutations identified in study samples from Vietnam (2000-2016). Results are presented as the number of mutations (n) out of total number of samples with a valid result (N).

K13 mutation	All			2000-2005			2006-2010			2011-2012			2013-2014			2015-2016		
	n	N	%	n	N	%	n	N	%	n	N	%	n	N	%	n	N	%
P443L	3	578	0.5%	0	67	0%	0	77	0%	3	238	1.3%	0	123	0%	0	73	0%
C469Y	8	578	1.4%	0	67	0%	0	77	0%	3	238	1.3%	0	123	0%	5	73	7%
T474I	26	578	4.5%	2	67	3%	0	77	0%	21	238	8.8%	3	123	2.4%	0	73	0%
G484V	2	564	0.4%	0	60	0%	0	75	0%	2	237	0.8%	0	120	0.0%	0	72	0%
**Y493H**	9	564	1.6%	0	60	0%	0	75	0%	5	237	2.1%	4	120	3.3%	0	72	0%
Y511H	4	564	0.7%	0	60	0%	0	75	0%	0	237	0%	0	120	0%	4	72	6%
**R539T**	15	564	2.7%	0	60	0.0%	1	75	1%	2	237	0.8%	12	120	10.0%	0	72	0%
**I543T**	149	564	26.4%	11	60	18.3%	28	75	37.3%	97	237	40.9%	10	120	8.3%	3	72	4.2%
**P553L**	35	561	6.2%	8	60	13.3%	0	76	0%	11	235	4.7%	11	118	9.3%	5	72	6.9%
V568G	2	561	0.4%	1	60	1.7%	0	76	0%	0	235	0%	1	118	0.8%	0	72	0%
**C580Y**	85	561	15.2%	3	60	5.0%	6	76	7.9%	13	235	5.5%	20	118	16.9%	43	72	59.7%
D584V	2	561	0.4%	0	60	0%	0	76	0%	0	235	0%	2	118	1.7%	0	72	0.0%
K607E	7	561	1.2%	1	60	1.7%	0	76	0%	5	235	2.1%	0	118	0%	1	72	1%

Supplementary Table S7. Drug resistance markers and Day 3 positivity rate after dihydroartemisinin-piperaquine (DHA-PPQ) treatment. The frequency of drug resistance markers to DHA (K13) and PPQ (*pm2* multiple copies) plus marker of resistance to mefloquine (*mdr1* multiple copies) are presented as the number of mutant (n) out of total number of samples with a valid result (N). Differences in the frequency of drug resistance markers by microscopically-detected parasitemia at Day 3 were determined using Chi-2 or Fisher's exact tests. The total sample size was 118, of which 85 corresponded to Quang Nam trial in 2011-2012 and 33 to Gia Lai trial in 2015-2016.

gene	mutation	Day 3 -			Day 3 +			*P*-value
		n	N	%	n	N	%	
**Markers of resistance**								
K13 (PF3D7_1343700)	Any ART-R validated SNP	46	62	74%	33	37	89%	0.119
pm2 (PF3D7_1408000)	>1 copy	3	56	5%	1	17	6%	1.000
mdr1 (PF3D7_0523000)	>1 copy	3	55	5%	1	17	6%	1.000
**ART-R parasite genetic background:**								
arps10 (PF3D7_1460900)	127M	54	61	89%	35	38	92%	0.737
fd (PF3D7_1318100)	193Y	49	56	88%	35	35	100%	0.041
crt (PF3D7_0709000)	326S	23	27	85%	20	23	87%	1.000

Supplementary Table S8. Complexity of infection (COI) in study samples from Vietnam (2000-2016). Data shows the number of single clone infections (*i.e.* COI=1; n) out of total number of samples with a valid result (N). COI was determined from 4 microsatellites in *pf*TSCA (542 samples) or a 101 SNP barcode by MassArray (MalariaGEN SpotMalaria, 468 samples).

		**2000-2005**			**2006-2010**			**2011-2012**			**2013-2014**			**2015-2016**			**p-value***
		**n**	**N**	**%**	**n**	**N**	**%**	**n**	**N**	**%**	**n**	**N**	**%**	**n**	**N**	**%**	
REGION 1	MS	(no data)			19	58	32.8%	41	115	35.7%	-	-	-%	10	49	20.4%	0.153
	SNP barcode	(no data)			22	27	81.5%	100	107	93.5%	6	6	100.0%	52	53	98.1%	0.065
REGION 2	MS	11	49	22.4%	3	23	13.0%	7	82	8.5%	1	36	2.8%	-	-	-%	0.029
	SNP barcode	38	39	97.4%	15	17	88.2%	73	80	91.3%	42	45	93.3%	-	-	-%	0.489
REGION 3	MS	0	16	0.0%	-	-	-%	3	26	11.5%	13	70	18.6%	3	18	16.7%	0.276
	SNP barcode	14	17	82.4%	-	-	-%	9	9	100.0%	51	54	94.4%	11	14	78.6%	0.090
All regions	MS	11	65	16.9%	22	81	27.2%	51	223	22.9%	14	106	13.2%	13	67	19.4%	0.136
	SNP barcode	52	56	92.9%	37	44	84.1%	182	196	92.9%	99	105	94.3%	63	67	94.0%	0.327

MS, microsatellites

Supplementary Table S9. Discriminant Analysis of Principal Components (DAPC) allele loadings. The top contributing alleles in the first 4 components (DA axis) of the DAPC analysis (see Figure 5) are listed with the value of the contribution of each allele to the variation in the corresponding axis.

position	allele	loading value	DA axis
Pf3D7_12_v3_1934745	G	0.038	1
Pf3D7_04_v3_891732	A	0.031	1
Pf3D7_12_v3_1934745	A	0.028	1
kelch_i543t	mutant	0.024	1
Pf3D7_01_v3_180554	G	0.024	1
Pf3D7_14_v3_2625887	G	0.023	1
Pf3D7_14_v3_1757603	A	0.023	1
Pf3D7_07_v3_619957	G	0.020	1
kelch_i543t	WT	0.019	1
Pf3D7_01_v3_535211	C	0.017	1
Pf3D7_07_v3_1358910	A	0.016	1
Pf3D7_09_v3_1379145	A	0.016	1
Pf3D7_04_v3_891732	C	0.014	1
kelch_t474_a	WT	0.014	1
Pf3D7_03_v3_656861	T	0.014	1
Pf3D7_13_v3_159086	A	0.013	1
kelch_t474_a	mutant	0.012	1
Pf3D7_14_v3_3046108	C	0.046	2
Pf3D7_14_v3_438592	A	0.042	2
Pf3D7_14_v3_438592	C	0.032	2
Pf3D7_14_v3_3046108	T	0.031	2
Pf3D7_11_v3_1815412	C	0.029	2
Pf3D7_11_v3_1815412	G	0.027	2
Pf3D7_13_v3_159086	G	0.023	2
Pf3D7_12_v3_2171901	T	0.022	2
Pf3D7_02_v3_376222	G	0.021	2
Pf3D7_06_v3_574938	C	0.020	2
Pf3D7_08_v3_701557	G	0.018	2
Pf3D7_11_v3_1018899	T	0.031	3
Pf3D7_14_v3_1757603	A	0.029	3
Pf3D7_11_v3_1815412	C	0.028	3
Pf3D7_11_v3_1815412	G	0.028	3
Pf3D7_11_v3_1802201	A	0.026	3
Pf3D7_01_v3_145515	A	0.024	3
Pf3D7_09_v3_452690	A	0.022	3
Pf3D7_06_v3_574938	C	0.021	3

Pf3D7_11_v3_1802201	G	0.020	3
Pf3D7_01_v3_145515	T	0.019	3
Pf3D7_06_v3_574938	A	0.018	3
Pf3D7_11_v3_1018899	C	0.016	3
kelch_c580y	mutant	0.015	3
Pf3D7_03_v3_155697	A	0.028	4
Pf3D7_03_v3_155697	G	0.023	4
Pf3D7_07_v3_1213486	G	0.022	4
Pf3D7_11_v3_477922	T	0.021	4
Pf3D7_13_v3_2161975	A	0.020	4
Pf3D7_12_v3_858501	A	0.018	4
Pf3D7_02_v3_839620	T	0.018	4
Pf3D7_13_v3_2161975	T	0.018	4
Pf3D7_07_v3_619957	G	0.017	4
Pf3D7_10_v3_1386850	T	0.017	4
Pf3D7_07_v3_1308383	C	0.016	4
