# Supplementary material for: Molecular surveillance of Plasmodium falciparum drug-resistance markers in Vietnam using multiplex amplicon sequencing (2000–2016)
Source: Sci Rep. 2023 Aug 25;13:13948. doi: 10.1038/s41598-023-40935-7 (PMC10457381; doi:10.1038/s41598-023-40935-7)
Supplement: Supplementary file 2 — Supplementary Information 2. [file 41598_2023_40935_MOESM2_ESM.pdf]

Molecular surveillance of *Plasmodium falciparum* drug-resistance markers in Vietnam using multiplex amplicon sequencing (2000-2016)

Supplementary Methods

Contents

Supplementary Methods………………………………………………………………………. 2
Design of the amplicon sequencing panel…………………………………………………………. 2
Selective whole genome amplification (sWGA) …………………………………………………. 2
Library preparation and sequencing……………………………………………………………… 3
Sequence data analysis……………………………………………………………………………… 4
Genotyping by mass spectrometry and 101-SNP molecular barcodes…………………… 4
Complexity of Infection…………………………………………………………………………… 5
Population genetics analysis………………………………………………………………………. 5
References…………………………………………………………………………………………… 6

Lab protocol: Multiplex amplicon sequencing of *P. falciparum* drug resistance markers using pfTSCA assay……………………………………………………………………. 8

Supplementary Methods

Design of the amplicon sequencing panel. SNP targets were selected for their reported association with antimalarial drug resistance in South-East Asia at time of experimental design (June 2017) and included: K13 as a marker of ART-R (full-length gene) [1], exonuclease codon E415G as marker of PPQ resistance[2], *crt* codons 72-76 as markers of chloroquine resistance [3], *mdr1* codon Y184F -the most frequent *mdr1* SNP in SEA- as a modulator of resistance to multiple antimalarials [3,4], as well as potential genetic modulators of ART-R *arps10*-V127M, *fd*-D193Y, *crt*-N326S, *mdr2*-T484I, MAL10:688956 and MAL13/RAD5-homolog-S1158A [5–8]. Genetic diversity was characterized using four microsatellite markers (*poly-α*, ARAII, TA81 and *pk2*) selected based on their high expected heterozygosity (He) level for Vietnam and their frequent use as markers for population surveillance in past studies [9,10]. Reference genomic sequences for each gene (*P. falciparum* 3D7 PlasmoDB build 29, June 2015) were submitted to Illumina Concierge Service (San Diego, United States) for the *in silico* design of an oligonucleotide probe panel adapted to sequencing chemistry of Illumina's TruSeq Custom Amplicon (TSCA, Illumina) method for ≈250bp-length fragments, prioritizing coverage of the whole K13 propeller domain and validated markers of resistance. Probes hybridizing upstream and downstream the selected SNPs could be designed for all major targets except *mdr2*-T484I and *exonuclease*-E415G resulting in a total of 21 amplicons for 11 genes (see Supplementary Tables S1 and S2). The assay covered the whole propeller domain of K13 and 73% of the upstream sequence between codons 1-440 (see Supplementary Fig. S2).

Selective whole genome amplification (sWGA). DNA samples were subject to *P. falciparum* sWGA by adapting the previously published protocol by Oyola *et al* [11]. Briefly, 50 µl reaction mixes were prepared containing 1.25x *phi29* buffer (New England Biolabs), 1.25x BSA (New England Biolabs), 2.5 µM of Pf1-Pf10 amplification primers [11], 1mM dNTP and 30 units of using *phi29* DNA polymerase (New England Biolabs) and 30 µl of DNA eluate (or 30 µl of nuclease-free water for negative controls run alongside samples). A

stepdown protocol was run in a thermocycler (35°C for 5 min, 34°C for 10 min, 33°C for 15 min, 32°C for 20 min, 31°C for 30 min, 30°C for 20 h) followed by a heat inactivation step of *phi29* enzyme at 65°C for 10 min and a cool down to 10°C. sWGA products were purified in 96-well plates using 1.8 volumes of Agencourt AMPure XP beads (Beckman Coulter) per 1 volume of sample, mixed by pipetting and incubated 15 min at room temperature. Beads were captured with a magnet and washed twice with 80% ethanol. Clean amplified DNA was subsequently eluted with 45 µl of TruSeq Resuspension Buffer (Illumina), supernatant transferred to a new plate and kept at 4ºC until library preparation on the following day.

Library preparation and sequencing. Purified *P. falciparum*-enriched DNA was quantified using Qubit HS DNA Assay Kit (Life Technologies) in a Qubit 2.0 fluorometer (Invitrogen), and samples were adjusted to 25-30 ng/µl with Resuspension Solution 1 (Illumina) when necessary. Libraries were prepared using TruSeq Custom Amplicon Low Input Library Prep Kit (Illumina), following manufacturer's guidelines for 96 samples. Hybridization of custom oligonucleotides program was run in a Veriti thermocycler (Applied Biosystems). After washing of unbound oligos and extension-ligation, products were indexed (i5 and i7) and PCR amplified (95°C for 3 min, followed by 33 cycles at 98°C for 20 sec, 67°C for 20 sec, 72°C for 40 sec, and final 72ºC for 1 min). A 5µl aliquot of purified PCR products was run in 2% agarose gel to confirm the correct amplicon size of 380-400bp. Each library was quantified using KAPA quantification kit for LightCycler 480 (Roche) and normalized to 5nM. All samples in a 96-well plate were pooled and stored at -20ºC. Prior to sequencing, pools were thawed, re-quantified using Qubit and adjusted to 4nM with Tris HCl according to the formula: (concentration in ng/µl / (660 g/mol x 380bp)) x 10^6. Libraries were denatured with an equal volume of 0.2N NaOH to a loading concentration of 13pM. Sequencing was conducted at Centre for Medical Genetics (University of Antwerp, Edegem, Belgium) in a MiSeq instrument with MiSeq Reagent Kit v2 (Illumina) and paired-end sequencing of 2x150bp reads.

Sequence data analysis. Demultiplexing, alignment and variant calling were performed using TruSeq Amplicon Workflow in MiSeq Reporter software (Illumina). The genome sequence of *P. falciparum* 3D7 PlasmoDB build 29 was used as reference. Variants were identified using Genome Analysis Toolkit (GATK, Broad Institute) and a variant filter quality cut-off score of 30. Individual VCF files were combined with Picard tools (Broad Institute), and the allele frequency and depth information were summarized in Excel spreadsheets. Genome Browse (Golden Helix) was used for visualization. Read depth cut-off was set at the maximum number of mapped reads for each amplicon found in results from 12 negative controls to control for spurious amplification. Mutations were only reported if found in at least two samples, or if the read count for the alternative allele was above the read cut-off for that amplicon. Loci with missing calls in more than 50% of all samples and controls were excluded. Haplotypes were built from calls with a ≥75% within-sample allele frequency to minimize risk of confounding by complex infections. Two amplicons (*k13.i* and *MAL10*) were sequenced in less than 50% of positive controls and were excluded from further analysis (see Supplementary Table S2). Median read depth of positive controls for the remaining amplicons in the assay was 7074x (medians range 34-27805x (Supplementary Table S2). Sensitivity for minor allele calling was estimated using three combinations of *P. falciparum* strains with known drug-resistance genotypes (3D7, wild-type reference; Cam1251, K13-C580Y and *arps10*-V127M; Dd2, *fd*-D193Y and *crt*-CVIET) at the following mix ratios: 3D7:Cam1251 at 90:10, 95:5 and 99:1; b) 3D7:Dd2 at 50:50, 80:20, 90:10, 95:5 and 99:1; c) Dd2:Cam1251 at 90:10. Both K13-C580Y and *arps10*-V127M variants were detected down to a 0.1 proportion, and *crt*-CVIET and *fd*-D193Y at up to 0.2 (see Supplementary Fig. S3). The latter was set as the minimum proportion for minor allele calling for all markers.

Genotyping by mass spectrometry and 101-SNP molecular barcode. DBS or DNA samples were shipped to Wellcome Sanger Institute (Cambridge, UK) for genotyping of drug resistance markers and 101 biallelic SNP barcode as part of MalariaGEN SpotMalaria Project (https://www.malariagen.net/projects/SpotMalaria). Mutations were identified by

mass spectrometry of PCR amplicons in a MassARRAY® System (Agena BioScience) as previously detailed elsewhere [12], except for *kelch*13-propeller domain which was analyzed by capillary sequencing. Results were provided in the form of a Genetic Report Card reporting amino acid changes at selected positions, except for SNP barcode result which was provided as a nucleotide sequence.

Complexity of infection. Complexity of infection (COI; *i.e*. the estimated number of genetically distinct parasites within the infection) was determined from MS-amplified regions with a separate alignment and variant calling procedure. Raw fastq files were aligned to MS-amplicon reference sequences (poly-alpha, TA81, ARAII and PfPK2) using Burrows-Wheeler aligner (v0.7.17) (141). Subsequently, reads in resulting bam files were realigned on repeats using Genotan v0.1.5 (147) and short tandem repeat (STR) length was determined using HipSTR (148). With HipSTR only the 2 most predominant MS genotypes (*i.e.* repeat lengths) present in the sequencing reads of a sample are determined. While this does not allow us to give exact estimates of COI by MS, we can distinguish between single clone (COI =1) vs. multiple clone infections (COI≥2), if 2 MS alleles are found for one or multiple MS markers. COI from SNP barcodes was determined using The Real McCOIL program [13].

Population genetics analysis. Expected heterozygosity (He) was calculated using the adegenet R package [14]. Genetic differentiation between populations (F_{ST}) was calculated using 1000 bootstraps with the package diveRsity [15]. Principal Component Analysis (PCA) was performed on the genotype matrix using ‘prcomp’ function (R stats package v4.0.5). Prior to PCA, missing genotypes were replaced by the mean allelic frequency at a locus in all samples. Discriminant Analysis of Principal Components (DAPC) was performed using adegenet with 40 principal components (PCs) and 11 discriminants (determined through cross-validation) to visualize the differentiation between predefined populations.

References

1. Ariey, F. *et al.* A molecular marker of artemisinin-resistant Plasmodium falciparum malaria. *Nature* **505**, 50–55 (2013).
2. Amato, R. *et al.* Genetic markers associated with dihydroartemisinin–piperaquine failure in Plasmodium falciparum malaria in Cambodia: a genotype–phenotype association study. *Lancet Infect Dis* **17**, 164–173 (2017).
3. Srimuang, K. *et al.* Analysis of anti-malarial resistance markers in pfmdr1 and pfcrt across Southeast Asia in the Tracking Resistance to Artemisinin Collaboration. *Malar. J.* **15**, 1–12 (2016).
4. Veiga, M. I. *et al.* Globally prevalent PfMDR1 mutations modulate Plasmodium falciparum susceptibility to artemisinin-based combination therapies. *Nat. Commun.* **7**, (2016).
5. Miotto, O. *et al.* Genetic architecture of artemisinin-resistant Plasmodium falciparum. *Nat Genet* **47**, 226–234 (2015).
6. Spring, M. D. *et al.* Dihydroartemisinin-piperaquine failure associated with a triple mutant including kelch13 C580Y in Cambodia: an observational cohort study. *Lancet Infect Dis* **15**, 683–691 (2015).
7. Takala-Harrison, S. *et al.* Genetic loci associated with delayed clearance of Plasmodium falciparum following artemisinin treatment in Southeast Asia. *Proc Natl Acad Sci U S A* **110**, 240–245 (2012).
8. Takala-Harrison, S. *et al.* Independent emergence of artemisinin resistance mutations among Plasmodium falciparum in Southeast Asia. *J Infect Dis* **211**, 670–679 (2015).
9. Anderson, T. J. C., Su, X. Z., Bockarie, M., Lagog, M. & Day, K. P. Twelve microsatellite markers for characterization of Plasmodium falciparum from finger-prick blood samples. *Parasitology* **119**, 113–125 (1999).
10. Hoffmann, E. H., Ribolla, P. E. & Ferreira, M. Genetic relatedness of Plasmodium falciparum isolates and the origin of allelic diversity at the merozoite surface protein-1 (MSP-1) locus in Brazil and Vietnam. *Challenges using Geogr. Inf. Syst. to*

understand Control Malar. Indones. **2**, 24–32 (2003).

11. Oyola, S. O. *et al.* Whole genome sequencing of Plasmodium falciparum from dried blood spots using selective whole genome amplification. *Malar J* **15**, 597 (2016).
12. Rovira-Vallbona, E. *et al.* Efficacy of dihydroartemisinin/piperaquine and artesunate monotherapy for the treatment of uncomplicated Plasmodium falciparum malaria in Central Vietnam. *J Antimicrob Chemother* **75**, (2020).
13. Chang, H.-H. *et al.* THE REAL McCOIL: A method for the concurrent estimation of the complexity of infection and SNP allele frequency for malaria parasites. *PLOS Comput Biol* **13**, e1005348 (2017).
14. Jombart, T. adegenet: a R package for the multivariate analysis of genetic markers. *Bioinformatics* **24**, 1403–1405 (2008).
15. Keenan, K., Mcginnity, P., Cross, T. F., Crozier, W. W. & Prodöhl, P. A. diveRsity: An R package for the estimation and exploration of population genetics parameters and their associated errors. *Methods Ecol. Evol.* **4**, 782–788 (2013).

Lab protocol: Multiplex amplicon sequencing of *P. falciparum* drug resistance markers using *pf*TSCA assay

Eduard Rovira-Vallbona, Pieter Guetens, Eline Kattenberg, Anna Rosanas-Urgell

Version: 1.2; 08/02/18

This protocol describes procedures for the multiplex amplicon sequencing of *Pf* drug resistance markers using Truseq Custom Amplicon Low Input Library Prep Kit (Illumina). This protocol should be used together with Illumina's TruSeq Custom Amplicon Low Input Kit Reference Guide: https://emea.support.illumina.com/downloads/truseq-custom-amplicon-low-input-library-prep-reference-guide-1000000002191.html.

Before starting:

- This protocol is designed as a 4-day workflow but can be divided into multiple days using safe stopping points or shorten it. Plan the whole process before starting.
- Prepare the list of samples to be tested and plate design for the different steps of the process. Consider space required for negative and positive controls, as well as internal controls of each experimental step. A maximum of 92 study samples is recommended.

DAY 1. Selective Whole Genome Amplification (sWGA) – est. time: 1.5h

Selective Whole Genome Amplification (sWGA) contributes to the untargeted amplification of *Plasmodium falciparum* genetic material from a human-*Pf* DNA mixtures or from low content *Pf* DNA samples, to increase *Pf* DNA input amount. This sWGA procedure was adapted from Oyola *et al,* Malar J, 2016 by increasing time to 20 h, and is an optional step. Application of sWGA may improve yield and sequencing performance in older samples.

- Thaw DNA samples (preferentially in ice) and keep at 4ºC.
- At pre-PCR area, thaw mastermix components in ice and prepare mastermix recipe for 50 µl sWGA reaction volume as follows:

**Add in order**	**n=1**	**n=96**
10x Phi29 buffer	6.25	625
100x BSA	0.625	62.5
250 µM primers	0.5	50
10 mM dNTP	5	500
Nuclease-free water	6	600
30units Phi29 Enzyme	3	300
Total volume	20 µl	*2137.5*

- Distribute 20µl mastermix per well.

- Add 30 µl of DNA sample to each well of sWGA plate.
- Add 30 µl of water as sWGA negative control.
- Cover with cap strips
- Briefly centrifuge (<1800 rpm, 1 min)
- Run sWGA program (22 h)

1. Step-down protocol<br>35ºC for 5 min,<br>34 ºC for 10 min,<br>33 ºC for 15 min,<br>32 ºC for 20 min,<br>31 ºC for 30 min,<br>30 ºC for 20 HRS<br>*2.Heat-inactivation of the Phi29*<br>65°C for 10 min<br>*3.Cool down at 10ºC until ready to remove*

DAY 2. Purification of sWGA products – est. time1.5h

The objective of this procedure is to clean up sWGA amplification products using magnetic beads. This step should be conducted in a separate area.

- Bring AMPureXP beads, samples, and Resuspension buffer (Illumina) to room temperature (allow for at least 30 min after you take out of the fridge). TE buffer can be used alternatively to Resuspension buffer.
- Prepare fresh 80% ethanol (400 µl/sample).
- Mix and vortex the bottle containing beads.
- Add 1.8V of beads per well using a repeater pipette (ex. for DNA volume of 40ul, add 72 µl)
- Transfer 40 µl of sWGA samples to the PCR plate.
- Using a manual multichannel pipette, mix by pipetting 10 times (try to avoid bubble formation).
- Cover with adhesive film and incubate 15 min at RT.
- Place the plate on a magnet and wait at least 5 min. Do not remove from magnet until indicated.
- Aspirate supernatant and discard.
- Add 180 µl of 80% fresh ethanol at RT, incubate 1 min.
- Remove ethanol and repeat procedure.
- Let residual ethanol evaporate for at least 15-20min.
- Remove plate from magnet.
- Add 45 µl Illumina Resuspension buffer and incubate 2 min.

- Mix well by pipetting using multichannel.
- Place plate on magnet and wait 5 min.
- Transfer supernatant to new clean plate. Store at 4ºC.

From this point, follow detailed procedures in *TruSeq Custom Amplicon Low Input Reference Guide*. Only modifications to the protocol are:

- Use Microseal A films to cover plates during centrifugation or shaking. Use strip caps for thermocycler incubations.
- Allow reagents in bead cleaning procedures to reach room temperature for at least 30min. Vortex and mix well several times, including in-between pipetting steps.
- For all incubations leave the plate in the magnet for 5 min. Use an elastic rubber to hold the plate in magnet.
- All ethanol solutions should be freshly prepared.

DAY 3. Library Preparation (quantification, hybridization, extension-ligation, amplification) – est. time full day

- Program the Hybridization program in the Veriti thermocycler before starting (please refer to Illumina's Reference Guide for programs in alternative thermocyclers)
- Clean lab bench and all materials with a DNA decontamination solution (DNA Zap) and wipe out with water.
- Identify the necessary reagents for hybridization step and leave them aside.
- Measure the ng/µl of purified products and other samples to be used in library preparation using Qubit HS DNA kit.
- If necessary, dilute DNA in RS1. As a general rule, add samples at 25-30 ng/µl.
- In a 96 well plate, load 2 µl of SS1 to each well and mix with 8 µl of each (diluted) sample, according to library plate layout.
- Follow 'Hybridize oligo pool' in Reference Guide and run HYB program in Veriti thermocycler as follows: (duration 2h)

ABI Veriti				
Lid temp 100°C				
Step	Ramp Speed	Increment (°C)	Temp (°C)	Hold
Step 1			95	3 mins
Step 2	2.1%	-0.5	90	30 sec
Step 3	Go to step 2 for 59x			
Step 4	2.0%	-0.5	60	1 min
Step 5	Go to step 4 for 19x			
Step 6	2.1%	-1.0	50	2 mins
Step 7	Go to step 6 for 9x			
Step 8	2.0%		40	10 mins

- Follow 'Remove Unbound Oligos' in Reference Guide. After final wash, let beads dry until "cracks" are observed". Do not remove the plate from magnet in the last step.
- Follow 'Extend and Ligate' in Reference Guide. Add ELB/ELE, and then remove plate from magnet. After adding ELB/ELE to DNA mixture, allow for 5 min before using pipette to resuspend.
- 'Amplify libraries' as in Reference Guide O/N, with cycles set as follows:

95°C for 3 minutes	
	98°C for 20 seconds
33X	67°C for 20 seconds
	72°C for 40 seconds
72°C for 1 minute	
Hold at 10°C	

DAY 4. Libraries clean-up, library quantification using KAPA kit, normalization and pooling – est. time full day

- Prepare 2% agarose gel with SYBRsafe (4 µl per 100 ml agarose), and let it cool down for later.
- Clean up libraries as in Reference Guide.
- Quality control of libraries:
    - With remaining liquid in CLP plate, carefully transfer 3ul to a PCR plate for post-PCR.
    - Add 2 µl of Loading Buffer
    - Load 5 µl in the agarose gel (include 100 bp marker) and run for 60min at 100V.
    - Targeted products should have a length of 380-400 bp.

- Store LNP plate at -20ºC until library quantification and sequencing (or proceed to next step).

The library quantification using KAPA kit starts here. Procedures are based on KAPA Technical Data Sheet (reference list). Note that two plates will be necessary to quantify a full libraries plate. After loading the first qPCR plate, start with the second plate dilutions.

- If frozen, thaw LNP plate.
- Prepare 300 ml of TrisHCl 10 mM pH8 from Tris stock using nuclease-free water
- Create a Kapa qPCR new plate design for 48 samples + 5 standards (in duplicate) + negative control (in duplicate)**.**
- Select some samples from LNP plate and use 1 µl to measure DNA concentration in Qubit or Nanodrop (if continuing from Day 4, you can also use 1 µl leftover in CLP plate)
- Estimate appropriate dilution for KAPA qPCR (normally 1/100.000 or 1/1.000.000 dilution should be ok)
- Dilute the first 48 samples in TrisHCl 10 mM pH8. Carefully check volumes when using multichannel (do not pipette less than 1 - 1.5 µl!)

    e.g. for a 1/100.000 dilution:

        Dilute 1.5 µl in 150ul Tris in a PCR plate. Mix well by pipetting 15x.
        Dilute 1.5 µl of previous step in 1500 µl Tris in a 2ml 96-weel plate.
        Mix well by pipetting slowly 15x.

- Prepare KAPA master mix for 48 samples + Standards + negative control (total n=62) in pre-PCR area, and aliquot into an appropriate plate for LC480.
- Load 4ul of Standards provided in the kit, negative controls, and diluted samples into qPCR plate.
- Briefly centrifuge and run KAPA program in LC480.
- Using qPCR results determine necessary dilution for each sample to obtain a 5nM concentration per well. Manually adjust dilution calculations to adapt volumes, if necessary.
- Dilute in TrisHCl 10 mM and label the diluted plate as SGP.

Start the 'Pool' libraries step by plate columns as in Reference Guide right after qPCR completion.

- Take 5µl of each row using multichannel and transfer to a 8-row PCR strip. Then mix the contents of each well into one single tube (label "PAL-RoundX 5 nM").
- Store left-overs of normalized SGP plate at -20ºC.
- Divide PAL tube in two aliquots and store at -20ºC, or proceed to the next step if sequencing is done in the next day.
- Perform PAL concentration adjustment to 4nM as close as possible to the sequencing run.

- Quantify the concentration in PAL-5nM tube using Qubit and estimate stock concentration in ng/µl. Apply the following formula to estimate concentration in nM:

$$\frac{concentration\ in\ ng/\mu l}{660\frac{g}{mol}\ \ x\ \ 380bp}\ \ x\ \ 10^{\wedge}6$$

- Readjust with Tris HCl to a final concentration of 4 nM.
- Store PAL 4nM at -20 ºC (at 4ºC if sequencing is conducted on the same day).

Sequencing requires the denaturation of library pools (refer to 'MiSeq System Denature and Dilute Libraries Guide') and preparation of a sample sheet following Illumina guidelines, or recommendations your sequencing service. Optimal loading conditions in a MiSeq using v2 reagents was set at 13 pM concentration and 5% PhiX.

List of materials

Reagents:

- Phi29 polymerase 125 µl - NEB, M0269L
- dNTPs (10mM pool -2.5 mM each)
- sWGA primer pool (250µM – 25µM each). See Oyola *et al*, Malar J, 2016 and MalariaGEN Resources https://www.malariagen.net/resources/amplicon-sequencing-toolkit/p-falciparum-amplicontoolkit-protocols
- Agencourt® Ampure® XP beads – Beckman Coulter
- Qubit dsDNA HS kit (ThermoFisher)
- Truseq Custom Amplicon Low Input Library Prep Kit 96 samples – Custom Design v1_1FC-134-2001
- TruSeq Custom Amplicon Index Kit (96 indexes, 384 samples) – Illumina, FC-130-1003
- KAPA quantification kit for LC480 500Rx – Roche Diagnostics KK4854 - 07960298001
- MiSeq Reagent Kit v2 (300-cycles) – Illumina, MS-102-2002
- Tris-HCl
- SYBR Safe DNA Gel Stain (ThermoFisher)
- DNAZap (Invitrogen)
- Nuclease-free water
- Agarose

Consumables:

- Adhesive films Microseal type A
- Cap strips
- 96-well PCR plates

- 96-well plates (2 ml volume/well)
- Nuclease-free 1.5 ml tubes
- Falcon tubes 50 ml
- Filter pipette tips
- LightCycler 480 optical qPCR plates

Equipment

- Plate shaker (1800 rpm)
- Multichannel pipette 10 µl
- Multichannel pipette 100 µl
- Multichannel pipette 300 µl or 1000 µl
- Monochannel pipettes
- Repeater
- Qubit fluorometer
- DynaMag™-96 Side Magnet (Thermo Fisher), or equivalent
- Verity thermal cycler (ThermoFisher)
- LightCycler480
- Gel electrophoresis tray and equipment

References

- KAPA Library Quantification Kit Illumina® Platforms Technical Data Sheet. January 2017 (KAPA Biosystems).
- MalariaGEN Guidance: selective Whole Genome Sequencing (sWGA). https://www.malariagen.net/resources/amplicon-sequencing-toolkit/p-falciparum-amplicontoolkit-protocols
- MiSeq System Denature and Dilute Libraries Guide (Illumina). Document #15039740 v01. https://emea.support.illumina.com/downloads/prepare_libraries_for_sequencing_miseq_15039740.htm Accessed January 2016.
- Oyola *et al*, Malar J, 2016. Whole genome sequencing of Plasmodium falciparum from dried blood spots using selective whole genome amplification.
- TruSeq® Custom Amplicon Low Input Library Prep Reference Guide (Illumina). https://emea.support.illumina.com/downloads/truseq-custom-amplicon-low-input-library-prep-reference-guide-1000000002191.html Accessed March 2016
